# Circular RNA differential expression in blood cell populations and exploration of circRNA deregulation in pediatric acute lymphoblastic leukemia

**DOI:** 10.1038/s41598-019-50864-z

**Published:** 2019-10-11

**Authors:** Enrico Gaffo, Elena Boldrin, Anna Dal Molin, Silvia Bresolin, Annagiulia Bonizzato, Luca Trentin, Chiara Frasson, Klaus-Michael Debatin, Lueder H. Meyer, Geertruij te Kronnie, Stefania Bortoluzzi

**Affiliations:** 1 Department of Molecular Medicine, University of Padova, Padova, Italy; 2 Department of Pediatrics and Adolescent Medicine, Ulm University Medical Center, Ulm, Germany; 3 Department of Women’s and Children’s Health, University of Padova, Padova, Italy; 4 CRIBI Biotechnology Center, University of Padova, Padova, Italy

**Keywords:** Transcriptomics, High-throughput screening, Haematopoiesis, Acute lymphocytic leukaemia

## Abstract

Circular RNAs (circRNAs) are abundantly expressed in the haematopoietic compartment, but knowledge on their diversity among blood cell types is still limited. Nevertheless, emerging data indicate an array of circRNA functions exerted through interactions with other RNAs and proteins, by translation into peptides, and circRNA involvement as regulatory molecules in many biological processes and cancer mechanisms. Interestingly, the role of specific circRNAs in leukemogenesis has been disclosed by a few studies, mostly in acute myeloid leukemia. In this study, circRNA expression in B-cells, T-cells and monocytes of healthy subjects is described, including putative new circRNA genes. Expression comparison considered 6,228 circRNAs and highlighted cell population-specific expression and exon usage patterns. Differential expression has been confirmed by qRT-PCR for circRNAs specific of B-cells (circPAX5, circAFF3, circIL4R, and circSETBP1) or T-cells (circIKZF1, circTNIK, circTXK, and circFBXW7), and for circRNAs from intronic (circBCL2) and intergenic regions that were overexpressed in lymphocytes. Starting from this resource of circRNA expression in mature blood cell populations, targeted examination identified striking and generalized upregulated expression of circPAX5, circPVT1 and circHIPK3 in pediatric B-precursor acute lymphoblastic leukemia, and disclosed circRNAs with variable expression across cytogenetic subtypes.

## Introduction

Circular RNAs (circRNAs), transcripts in which a downstream splice donor site is covalently bound to an upstream acceptor site by backsplicing, were recently recognized as evolutionary conserved and particularly stable transcriptome elements expressed with cell type- and differentiation-specific patterns^1,2^.

In the last few years, diverse functional roles of specific circRNAs were discovered, making them attractive biological molecules for both fundamental and cancer research^3^. By acting as microRNA (miRNA) sponges and competitive endogenous RNAs, circRNAs can indirectly regulate miRNA-target expression, ultimately controlling key miRNA-involving axes^4,5^. CircRNAs can also interact with RNA-binding proteins^6^ and regulate cellular processes, as shown for circFOXO3, which controls cell cycle progression by binding p21 and CDK1^7^. Notably, translation of some circRNAs into peptides not encoded by linear transcripts was demonstrated^8–10^ and linked to circRNA function, clarifying for instance the role of circZNF609 in the regulation of myoblast differentiation^9^ and rhabdomyosarcoma progression^11^.

CircRNAs are abundantly expressed in the haematopoietic compartment^12,13^. Identification of “scrambled exons” not only in hyperdiploid B-cell acute lymphoblastic leukemia (B-ALL), but also in naïve B-cells, haematopoietic stem cells and neutrophils, showed that circRNAs are present both in normal and malignant haematopoietic cells^14^. Considering normal haematopoiesis, Maass and colleagues^15^ described ~3,700 circRNAs expressed in plasma, serum, neutrophils, and platelets. Interestingly, enrichment of circRNAs compared to the host gene linear isoforms was found in whole blood^12^, platelets and red blood cells^16^. Moreover, a recent work on the expression of 489 circRNAs across the haematopoietic tree showed 102 circRNAs differentially expressed in different cell types and maturation stages^17^ and prompted further investigation on circRNA expression variation in blood cell populations.

CircRNA involvement in cancer mechanisms is a prosperous research field^18^. Reports of circRNA expression variation in acute myeloid leukemia^19^, together with the deregulation of circRNAs expressed from MLL and its fusion partners in MLL rearranged leukemia^20,21^, pointed at a role of circRNAs in haematopoietic malignancies. Data on circRNA expression and function in lymphoid malignancies are still limited, but indicate participation of these molecules in the disease. CircPVT1 derives from *PVT1* gene, which resides close to *MYC* in 8q24, identified as a risk locus of many cancers including leukemia. The circular but not the linear transcript of *PVT1* has high expression specifically in acute lymphoblastic leukemia^22^. Dahl *et al*.^23^ provided the first information on the circRNAome in malignancies of mature B-cells, mantle cell lymphoma and multiple myeloma. In T-cell lymphoblastic lymphoma high circLAMP1 expression was recently linked to modulation of cell growth and apoptosis by regulation of the miR-615-5p/DDR2 pathway^24^.

This study aims at enriching the knowledge of circRNA expression variation in mature blood cell populations and contributing to the exploration of circRNA expression in acute lymphoblastic leukemia. Investigation of the circular transcriptome of B-cell, T-cell and monocyte populations, reconstructed from high-depth RNA-seq data, disclosed differential and cell type-specific circRNA expression and alternative circularization patterns. Starting from this resource, a targeted examination of a set of circRNAs identified deregulated expression in cytogenetic subtypes of pediatric B-cell precursor acute lymphoblastic leukemia (BCP-ALL).

## Results

### CircRNAomes of B-, T-cell and monocyte populations

CircRNA expression in B-, T-cell and monocyte populations of healthy donors was investigated using high-depth ribodepleted RNA-seq data of 12 samples, with 4 replicates for each population of cells sorted from peripheral blood mononuclear cells, PBMCs (GEO series ID: GSE110159; Supplementary Methods and Supplementary Table 1).

CircRNA quantification and annotation were provided by CirComPara^25^, which combined 9 circRNA detection software tools (CIRI2^26^ Findcirc^27^; CIRCexplorer2^28^ on BWA^29^, STAR^30^, Segemehl^31^ and TopHat2^32^ aligners; DCC^33^; circRNA_finder^34^; and Segemehl^31^) to obtain the most reliable backsplices. Indeed, shared output from two or more algorithms for circRNA detection has been demonstrated to reduce false positive predictions^35,36^. CirComPara performs read pre-processing quality filters, such as adaptor trimming, read mean quality selection and filtering by read length. Moreover, CirComPara counts the linearly spliced reads aligned to the backsplice junctions of each circRNA to estimate the expression of linear transcripts expressed from the circRNA’s host-gene. These values were combined with the backspliced read counts, which measure the circular expression, to compute the proportion of expression between the circular and linear isoforms (Circular to Linear expression Proportion, CLP; see Methods). Further, the CLP embeds the concept of circular to linear expression correlation, so that CLP variation across conditions conveys the rate of independence between a circRNA and its host-gene’s linear expression^33^.

Overall, 68,007 circRNAs from 10,148 individual genes were detected by at least two methods. As reported by Hansen *et al*.^36^, the algorithms mostly agreed on highly expressed circRNAs, whereas those detected by only one algorithm had generally low read counts (Supplementary Fig. 1).

Further, a sub-set of 6,228 circRNAs (from 3,323 genes) showing expression in all biological replicates of at least one cell type was retrieved and referred to as “high confidence” (HC) circRNAs (Supplementary Table 2). Comparison of circRNAs reported in this study with the results of Nicolet *et al*.^17^ confirmed concordance for 83% of the 489 HC circRNAs retrieved in the previous study and disclosed 5,824 additional HC circRNAs that were not yet investigated for expression variation in blood cell populations (Supplementary Fig. 2A).

**Figure 1:**
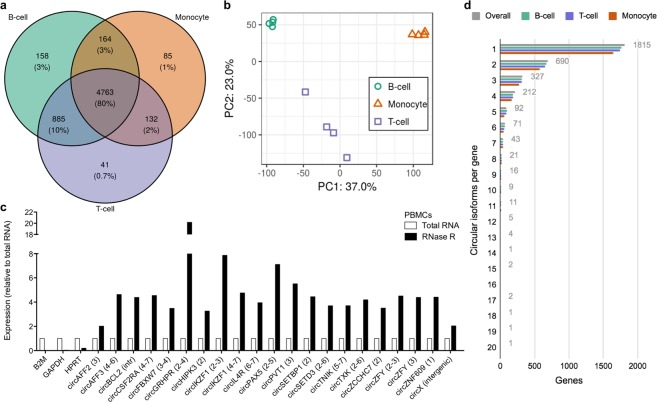
Comparison of circRNAome of B-, T-cells and monocytes. (**a**) Overlap of the 6,228 high confidence (HC) circRNAs expressed in B-, T-cells and monocytes; (**b**) Principal component analysis of HC circRNA expression profiles; (**c**) Validated circRNAs after RNase R treatment. B2M, GAPDH and HPTR mRNAs are shown as positive controls; Relative expression is calculated as 2^−∆Cq^, where ∆Cq is the difference between RNase R treated and total PBMC RNA; (**d**) Number of circRNAs per gene expressed overall, and in each cell type.

Of the 6,228 HC circRNAs, 5,970 and 5,821 were expressed in B- and T-cells, and 5,144 in monocytes (Fig. 1a). The majority of circRNAs (4,763; 80%) were detected in all three cell types, including ubiquitous circZNF609^37^, circHIPK3^38^ and novel circRNAs. New circRNAs (e.g. circPICALM) are likely to be specific for the haematopoietic compartment. CircRNAs shared by two cell types where mostly common between lymphocytes.

Unsupervised principal component analysis showed relatively small variation of circRNA expression profiles within replicates of the same cell population, and pointed at circRNAome differences that clearly discriminated the cell types (Fig. 1b).

Expression of 21 HC circRNAs was validated by RT-PCR in PBMCs of different healthy donors (Supplementary Table 3; Table 1; Fig. 1c). This validation included exonic circRNAs from 15 different genes, two alternative isoforms of *IKZF1* and of the male-specific *ZFY* from the Y chromosome, one circRNA (18:63280887-63281214:−) derived from circularization of 328 bp of the unique large *BCL2* intron, and one circRNA from a putative new gene (“intergenic”, see below). The circular structure of circRNAs was corroborated by the observed enrichment of circRNAs after RNase R treatment, and detected by qRT-PCR with divergent primers specific for the backsplice junction^14,27^. Moreover, all predicted backsplice junctions were confirmed by Sanger sequencing.

**Table 1:** Summary of validated circRNAs.

CircRNA	Expression				Previous data	
	Reads			qRT-PCR	CircRNA	Locus
	B	T	M			
circAFF2 (3) X:148661908–148662768:+	18	6	113	Upregulated in TCF3-PBX1 ALL	Downregulated in acute myeloid leukemia (AML) compared with healthy controls^72^; Upregulated in monocytes^17^	Deletion of the locus associated with Fragile X syndrome^73^; Linear transcripts not expressed
circAFF3 (4–6) 2:100006632–100008932−	93	0	5	Upregulated in B-cells and ALL	—	Involved in fusions with MLL in ALL^74^
circBCL2 (intr) 18:63280887–63281214:−	36	40	0	Upregulated in lymphocytes and ETV6-RUNX1 ALL	—	Involved in frequent translocations in follicular lymphoma^75^
circCSF2RA (4–7) X:1285778–1290509:+	1	0	186	—	—	Frequently deleted in BCP-ALL^76^; Linear transcripts not expressed
circFBXW7 (3–4) 4:152411303–152412529:−	595	1145	334	Upregulated in T-cells and expressed in ALL	Tumor suppressor in glioma^46^, clear cell renal cell carcinoma^77^ and breast cancer progression^78^ by miRNA sponging and protein coding potential.	Mutated or deleted in T-ALL^79^; Circular isoform higher than the linear form^80^
circGRHPR (2–4) 9:37424845–37426654:+	90	50	23	—	Validated in^10^	Involved in fusions with *BCL6* in some lymphomas^81^
circHIPK3 (2) 11:33286413–33287511:+	151	187	553	Upregulated in monocytes and ALL	Oncogenic in different cancer types^43^	Circular isoform more abundant than the linear one^38^
circIKZF1 (2–3) 7:50319048–50327757:+	77	211	30	Downregulated in T-cells, and in BCR-ABL1, hyperdiploid ALL	—	Deleted or mutated in ALL^82^
circIKZF1 (4–7) 7:50376533–50391863:+	11	20	13	—	—	
circIL4R (6–7) 16:27346467–27352696:+	154	13	0	Upregulated in B-cells, Downregulated in ALL	Deregulated expression in hepatocellular carcinoma^47^	Frequently mutated in primary mediastinal large B-cell lymphoma^83^
circPAX5 (2–5) 9:37002648–37020801:−	34	0	0	Upregulated in B-cells and ALL	—	Involved in translocations or deletions in haematologic malignancies^41^
circPVT1 (3) 8:127890589–127890998:+	18	20	3	Upregulated in ALL	Oncogenic in different cancer types^48^, Upregulated and oncogenic in ALL^22^	Oncogenic long non-coding RNA^84^, Upregulated and o ncogenic in T-ALL^85^
circSETBP1 (2) 18:44701175–44701832:+	136	13	5	Upregulated in B-cells and hyperdiploid ALL	—	Somatic mutations in myeloid leukemia^86^
circSETD3 (2–6) 14:99458279–99465813:−	201	367	402	—	Not binding to Argonaute^10^, Downregulated in colorectal cancer^87^, Tumor suppressor in hepatocellular carcinoma, sponge for microRNA-421^88^	Amplification (14q32) in AML^89^; Linear transcripts not expressed
circTNIK (5–7) 3:171188702–171194635:−	0	43	0	Upregulated in T-cells	Upregulated in quadriceps femoris muscle of myotonic dystrophy^90^	—
circTXK (2–6) 4:48104901–48114402:−	1	72	0	Upregulated in T-cells	—	—
circZCCHC7 (2) 9:37126312–37126942:+	1048	385	94	Upregulated in B-cells, Downregulated in ALL	Associated with polysomes^10^; Validated in^10^	Downregulated in relapsed ALL^91^ Linear transcripts not expressed
circZFY (2–3) Y:2953909–2961646:+	70	106	50	Upregulated in ALL	Validated in^38^, Increased in tuberculosis patients^92^	—
circZFY (3) Y:2961074–2961646:+	48	97	48	—	—	—
circZNF609 (1) 15:64499293–64500166:+	254	743	285	—	Roles in Hirschsprung disease (ceRNA)^37^, and in myoblast proliferation (protein coding)^9^; Validated in^37^	—
circX (intergenic) X:65051462–65075912:+	214	294	3	Upregulated in lymphocytes, Downregulated in ALL	—	—

Twenty-one circRNAs were validated by RT-PCR in PBMCs of healthy donors and all were resistant to RNase R treatment. CircRNA names include the gene locus, the exons involved in the backsplicing and the genomic coordinates. Expression (Reads) is indicated as the average read count of the four biological replicates per each cell type (B, B-cell; T, T-cell, M, monocyte). Expression (qRT-PCR) indicates circRNAs for which expression in blood cell populations and in BCP-ALL was screened by qRT-PCR. Previous data provide information about the circRNA and/or the locus from the literature.

### Multiple circular isoforms and circRNAs from new genes

Nearly all circRNAs (99.4%) derived from annotated genes, prevalently with backsplice junctions overlapping known exons (98.9%). Of the 71 circRNAs with both ends in intronic regions, the most abundant ones included the lymphocyte-specific circBCL2, which was validated, circHLA-E, circRASSF3, and several circMBL1 isoforms.

Almost one half of the circRNA host genes expressed multiple (up to 20) circular isoforms each (Fig. 1d). Preferential backsplice junction usage and expression of one or few prevalent isoforms were observed. The highest numbers of isoforms were expressed in monocytes by *AGTPBP1* (20) and *PICALM* (15), and in lymphocytes by *UBAP2* (19) and *ATM* (17).

Thirty-four circRNAs derived from intergenic regions. Intergenic circRNAs using the same backsplice ends in different combinations identified three loci expressing multiple isoforms. Five “intergenic” circRNAs derived from a putative new gene in the Xq11.2 region (chrX:65051462-65113813) (Supplementary Fig. 3). The most abundant circRNA of the locus, circX(intergenic) (X:65051462-65075912:+), previously detected in blood also by Memczak and colleagues^12^, was validated (Fig. 1c).

Next, we investigated to what extent the expression is in favor of circular with respect to linear transcripts overlapping the backsplice junctions. CLP values range from 0 to 1: 0 comes when no circular expression is detected, 0 < CLP < 0.5 represents circRNAs expressed less abundantly than the respective linear isoforms, 0.5 means that circular and linear transcripts have equivalent abundance, 0.5 < CLP ≤ 1 indicates circular isoforms expressed more abundantly than the respective linear transcripts. In particular, CLP = 1 when the linear expression relative to the circRNA is not detected. Interestingly, for 10 circRNAs no linear expression was detected. Moreover, the CLP was remarkably high (>0.95) for 14 circRNAs (Supplementary Table 4), including circGUSBP2 and circNBPF10, with median CLP ranging from 0.99 to 1 in all cell populations (Supplementary Table 4), and circAFF2, which showed high CLP in monocytes (0.97). Preferential transcript circularization in mature blood cells of specific genes^5,39^ and/or higher stability of circular compared to linear RNAs^16,40^ could explain these findings.

### Comparison between cell types disclosed cell type-specific circRNA expression and alternative circularization patterns

**Figure 2:**
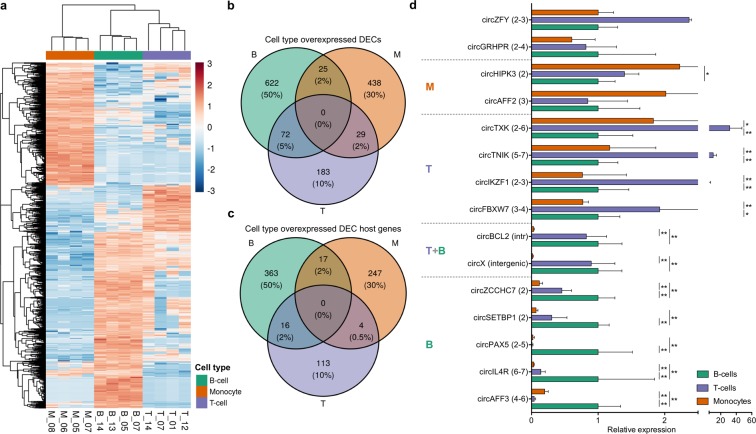
Differential expression of circRNAs in mature blood cell populations. (**a**) Hierarchical clustering (Euclidean distance; complete clustering) of expression profile of 1,369 significantly differentially expressed circRNAs (DECs) between cell types. (**b**) Venn diagram showing the overlap of circRNAs overexpressed in each population: non-intersection portions outline cell type-specific overexpressed circRNAs. (**c**) Host genes for DECs overexpressed only in one cell type: intersection regions represent genes expressing different circular isoforms overexpressed in different cell types. (**d**) qRT-PCR validation of expression of 15 circRNAs, 13 of which are significantly overexpressed in monocytes (M), T-cells (T), B-cells (B) or both lymphocyte populations (T + B). In agreement with RNA-seq data, circZFY and circGRHPR were not significantly differentially expressed. Gene exons involved in the backsplice are displayed in parentheses after the circRNA name. Expression relative to the mean of B-cells. Mann-Whitney U-test, p-value * < 0.05, ** < 0.01.

Next, we aimed to define differences of the B-, T-cell and monocyte population circRNAomes. Pairwise comparisons of the three populations identified overall 1,369 significantly differentially expressed circRNAs (DECs) between cell types (Supplementary Table 5), which derived from 880 genes. Hierarchical clustering of DEC expression profiles reflected the sets of circRNAs upregulated in each cell type (Fig. 2a). DECs exclusively or over-expressed in one cell type indicated population-specific circRNAs (Fig. 2b): 622 were characteristic of B-cells, 183 of T-cells, and 438 of monocytes (1,243 in total; Supplementary Table 5). Moreover, 72 DECs were upregulated in both lymphocyte populations (Fig. 2b–d). No significantly enriched KEGG pathways of gene ontology terms resulted for genes of B-cell-characteristic circRNAs, which nevertheless, included genes involved in the B-cell receptor signalling pathway, such as SOS2 and NFKB1, or linked to B-cell functions. On the contrary, genes expressing T-cell characteristic circRNAs were significantly enriched the T-cell receptor signaling pathway. Moreover, genes of monocyte-characteristic circRNAs significantly enriched several biological processes and pathways related to monocyte functions. Other cell type-characteristic host-genes, instead, had cell functions not directly linked to the cell of origin (Supplementary Table 6).

Although cell type-characteristic circRNA sets were disjoint, the overlap of gene sets that expressed specific isoforms indicated alternative cell type-specific circularization patterns. Of note, 37 genes expressed circRNAs characteristic of two cell types and accounted for 14.7% of the genes with multiple cell type-specific circRNAs (Fig. 2c). Four circAKT3 isoforms showed cell type-specific expression, three for B- and one for T-cells; also four circMBNL1 were cell type-specific, 3 for B-cells and one for monocytes. Moreover, six different GRK3 circular isoforms were specifically overexpressed in B-cells, whereas two others were overexpressed only in monocytes.

Quantification by qRT-PCR in B-cells, T-cells and monocytes sorted from 5 independent healthy donors confirmed RNA-seq results for all 15 tested circRNAs, supporting data robustness and reproducibility (Fig. 2d and Supplementary Fig. 4).

Significant up-regulation in B-cells of 5 circRNAs, including circAFF3 (exons 4–6), circIL4R (exons 6–7), circSETBP1 (exon 2) was confirmed. Moreover, high circRNA expression from the genomic region 9p13.2 including *PAX5* (Supplementary Fig. 5) was validated: circPAX5 (exons 2–5) and circZCCHC7 (exon 2) were both B-cell-specific, while a trend toward upregulation of circGRHPR in B-cells was in agreement with the estimate from the RNA-seq data. *PAX5* exerts a striking role in the commitment of B-cells^41^ and co-expression of *PAX5* and *ZCCHC7* linear transcripts was described during the progression from common lymphocyte precursors to pre-pro-B cells^42^. Suggestive of co-regulation of the 9p13.2 locus, circPAX5, circZCCHC7 and circGRHPR isoforms were all overexpressed specifically in B-cells. CircPAX5 and circZCCHC7 were previously detected in CD19 + cells^14^. Both circZCCHC7 and circGRHPR were identified in CD34 + cells and, according to data on RNA base modification promoting efficient initiation of protein translation from circRNAs in human cells, are likely to encode peptides^10^.

Regarding T-cells, significant overexpression was confirmed for circIKZF1 (exons 2–3), circTNIK (exons 5–7), circTXK (exons 2–6) and, in agreement with previous reports^17^, for circFBXW7 (exons 3–4). Also an increasing trend for circZFY (exons 2–3) in T-cells and for circAFF2 (exon 3) in monocytes, in agreement with Nicolet *et al*.^17^, confirmed RNA-seq results, while significant upregulation of circX(intergenic) and circBCL2(intronic) in lymphocytes and of circHIPK3 (exon 2) in monocytes was validated.

Nicolet *et al*.^17^ found 102 circRNAs differentially expressed across blood cell types and stages, 98 of which were detected in our data. In particular, 42 circRNAs resulted differentially expressed also in our comparison, 31 of which were cell type-specific. Overall, our data where in agreement with circRNA clusters previously associated to mature cell populations (Supplementary Fig. 2b). CircRNAs previously assigned to lymphoid cell-specific clusters showed the highest expression in B-cells or T-cells, including circZCCHC7 and circFBXW7 that we experimentally validated. Nine out of 10 circRNAs previously deemed as monocyte-specific were recalled by our analysis being more expressed in monocytes, including circAFF2. However, the majority of the 1,243 circRNAs defined as cell type-specific in the present study, including 11 out of 15 circRNAs for which cell type-specific overexpression was confirmed by qRT-PCR in this study (Fig. 2d), were not represented in the clusters defined by Nicolet *et al*.

Next, we inspected whether the abundance of circular isoforms with respect to linear expression was altered between the cell types. CLP variations across sample conditions indicate the rate of independence between a circRNA and the host-gene linear expression. First, we observed that the number of circRNAs with more abundant circular expression proportion (CLP > 0.5) was highest in lymphoid cells (185 in monocytes, 333 and 364 in B-cells and T-cells, respectively), which is in accordance with previous observations on a smaller set of circRNAs^17^. Then, we identified 687 circRNAs (from 495 genes) with host-gene independent expression (Supplementary Table 7). Among DECs, 163 had significant variation of circular expression proportion between cell types in agreement to the differential absolute expression, indicating that observed variations of these circRNA expression level across cell populations are not due to a corresponding variation of linear expression. CircIKZF1, for which upregulation in T-cells was validated, was also expressed with high CLP in T-cells. In particular, 25 circRNAs showed high and significantly varied circular expression proportion (Supplementary Fig. 6), including the validated circX(intergenic) and three additional intergenic circRNAs, all overexpressed in B- and T-cells. CircSMARCA5 had the highest absolute and relative circular expression in B-cells, while it was significantly lower in T-cell and lowest in monocytes (Supplementary Fig. 7).

### Expression of circRNAs in six cytogenetic subtypes of B-cell precursor acute lymphoblastic leukemia

Starting from the above described transcriptome-wide circRNA resource, the expression and possible deregulation of circRNAs in BCP-ALL was examined for a target set of circRNAs. The selected circRNAs showed lymphocyte specificity and/or derived from leukemia-associated loci. Following these criteria, ten of the circRNAs with validated upregulation in B-cells, T-cells or in both lymphocyte populations (Fig. 2d) were selected for quantification in BCP-ALL, including circRNAs from known genes (*AFF2*, *AFF3*, *BCL2*, *FBXW7*, *IKZF1*, *IL4R*, *PAX5*, *SETBP1* and *ZCCHC7*) and the newly identified circX(intergenic) highly expressed in lymphocytes. In addition, circZFY, a circRNA expressed at a high level in blood cells of male subjects; circHIPK3, for which oncogenic properties are known in solid cancers^43^; and circPVT1, recently linked to acute lymphoblastic leukemia^22^, were included.

Expression of the 13 selected circRNAs was measured by qRT-PCR in 32 BCP-ALL patient-derived xenograft (PDX) samples (Supplementary Table 8).

**Figure 3:**
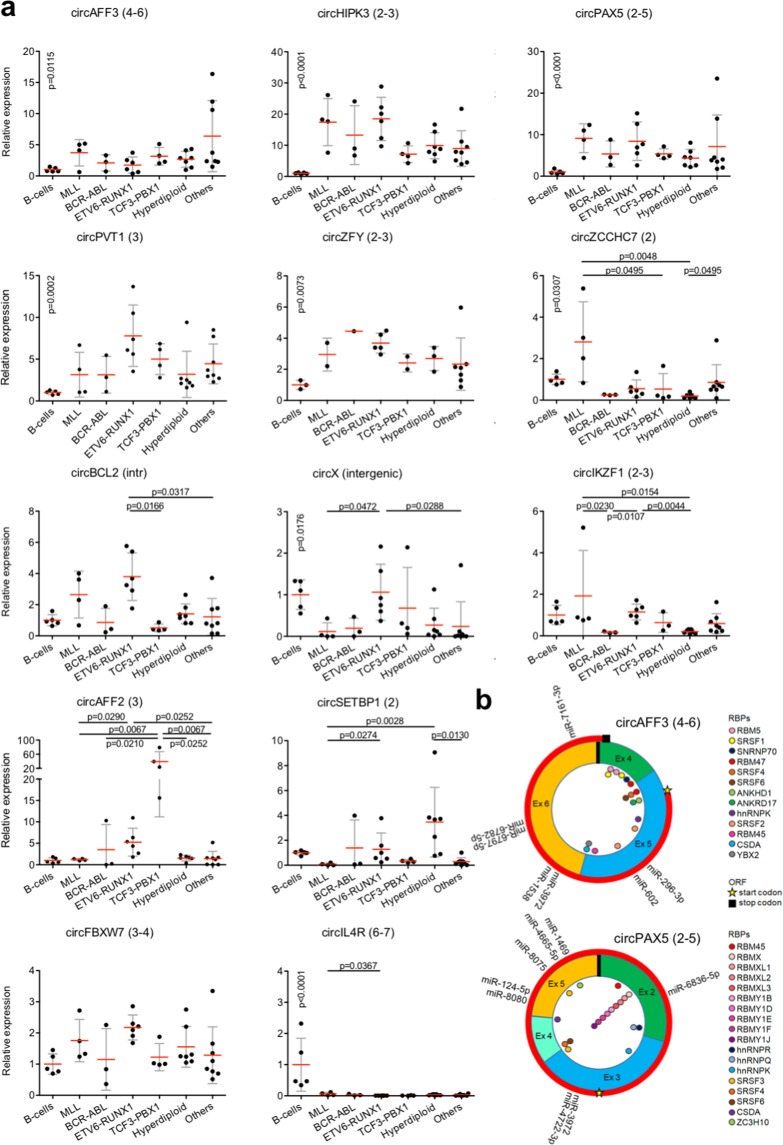
CircRNA expression in patient-derived BCP-ALL samples and predicted functions of circAFF3 and circPAX5. (**a**) Relative expression of 12 circRNAs assessed by qRT-PCR in healthy PBMC derived B-cells and 32 BCP-ALL patient-derived xenograft samples with different genetic subtypes: MLL rearranged (n = 4), BCR-ABL (3), ETV6-RUNX1 (6), TCF3-PBX1 (4), high hyperdiploid (>50 chromosomes, 7), “Others” (negative for the mentioned rearrangements, 8). Expression relative to B-cells. Only significant p-values (<0.05) are indicated (Kruskal-Wallis test, corrected for multiple testing with Benjamini, Krieger and Yekuteli procedure). P-values on “B-cells” refer to B-cells vs all BCP-ALL samples (Mann Whitney U-test, only significant values shown). (**b)** Prediction of functions and interactions of circAFF3 and circPAX5, including predicted miRNA-binding sites, sequence motifs possibly recognized by RNA binding proteins (RB) and open reading frames (ORFs) of at least 150 nt spanning the backplice.

All leukemic samples together were first compared with B-cells from healthy donors (Supplementary Fig. 8 and Fig. 3a) to check for deregulated circRNA expression in leukemic cells. For seven circRNAs the expression was significantly different in ALL samples compared with B-cells. CircIL4R, circZCCHC7 and circX(intergenic), all highly expressed in lymphocytes, were less expressed in ALL. Conversely, overexpression of circAFF3, circHIPK3, circPVT1 and circPAX5 in BCP-ALL emerged. Differently from circPVT1 and circHIPK3, a functional characterization of circPAX5 and circAFF3 is still lacking. Thus, custom functional predictions, in terms of possible miRNA- binding sites, RNA binding protein (RBP) binding sites, and coding potential were obtained (Fig. 3b and Supplementary Fig. 9).

Further, we explored expression of the target set of circRNAs across the main BCP-ALL cytogenetic subtypes (Fig. 3a). Cytogenetic subtypes are characterized by specific genetic lesions, including recurrent translocations (MLL rearrangements, BCR/ABL, ETV6-RUNX1, and TCF3-PBX1 fusions) and hyperdiploid karyotype. Cytogenetic subtype characterization is instrumental for risk prognosis and treatment stratification of leukemia patients. Leukemic cells of different subtypes have distinct biological features, gene expression profiles and specific miRNA signatures^44,45^. In this context, novel information about the heterogeneous nature of acute leukemias was added by the observed significant circRNA expression differences among cytogenetic subtypes (Fig. 3a). CircAFF2 was highly expressed in TCF3-PBX1 BCP-ALL and, to a lesser extent, in ETV6-RUNX1 BCP-ALL, compared to B-cells and other cytogenetic BCP-ALL subgroups. CircBCL2(intronic) was upregulated in ALL with ETV6-RUNX1 fusions. CircSETBP1 and circX(intergenic) were both very reduced in MLL rearranged samples. CircIKZF1 was lower in BCR-ABL and hyperdiploid leukemias compared with the ETV6-RUNX1 subtype, in which the expression was conserved at levels comparable with B-cells.

## Discussion

This study disclosed the circRNAomes of B-, T-cell and monocyte populations, first providing a large catalogue of circRNAs expressed in normal B-, T-cell and monocytes. Comparison of the cell population circRNA expression profiles confirmed previous findings on variable circRNA expression among different blood cells^17^ and further suggested hundreds of circRNAs with cell type-specific expression. Moreover, complex patterns of cell type-specific alternative circularization were disclosed. These results were backed up by qRT-PCR validations in independent samples for selected circRNAs.

We speculate that at least some of the circRNAs with high cell type-specific expression could be involved in the regulation or maintenance of the specific cell functions. Functional data were available only for a few of the circRNAs that we validated for specific overexpression, and when available, they were often from a different context (Table 1). For instance, circFBXW7, overexpressed in T-cells, was shown to suppress cancerogenesis and malignant progression in solid tumors, and to encode a 185 amino acids functional peptide^46^. Besides, circZCCHC7, highly expressed in lymphocytes, was shown to be associated to polysomes in 293 and HeLa cells^10^. Therefore, further functional experiments, like circRNA overexpression or knockdown, will be required to elucidate the role of specific circRNAs in normal haematopoiesis.

In a prospective view toward understanding translational relevance of our findings, the second part of the study reported deregulation of circRNAs in BCP-ALL disclosed by screening of a target set of circRNAs in six cytogenetic subtypes. Considering circRNAs dowregulated in BCP-ALL, circIL4R showed deregulated expression also in hepatocellular carcinoma^47^. CircX(intergenic), highly expressed in lymphocytes and less expressed in ALL, could derive from an unannotated gene that produces only circularized transcripts.

Moreover, four circRNAs were markedly upregulated in BCP-ALL: circPVT1, circHIPK3, circPAX5 and circAFF3. Observed circPVT1 upregulation in pediatric BCP-ALL is in line with previous reports of various cancer types^48^ including ALL^22^. CircPVT1, originating from a cancer susceptibility locus including the oncofoetal lncRNA PVT1 and MYC, was first detected in gastric cancer, where upregulation promotes cell proliferation by sponging members of the miR-125 family^49^. As recently reviewed^48,50^, functional studies showed circPVT1 oncogenic properties in solid tumours: circPVT1 sponges different tumour suppressor miRNAs and hence derepresses expression of oncogenic proteins, ultimately promoting tumor-associated phenotypes like proliferation, invasion, angiogenesis and drug resistance. Plus, circPVT1 levels significantly impact drug response in multiple myeloma^51^ highlighting its therapeutic potential. CircPVT1, but not PVT1 linear transcript, was shown to be upregulated in bone marrow of adult ALL patients^22^. Knockdown of circPVT1 in B- and T-ALL cell lines arrested cell growth and induced apoptosis through inhibition of c-Myc and Bcl-2 expression mediated by sponging of miR-125 and miR-let7. Recently, Tashiro *et al*.^52^ reported the potency of circPVT1 to encode a protein of 140 amino acids. Our data are the first observation of a general upregulation of circPVT1 in specimens of pediatric patients with BCP-ALL. In non-small cell lung cancer, Qin *et al*.^53^ showed that circPVT1 regulates Bcl-2 expression by sponging miR‐497. The tumour suppressor role of miR-497 in cancer entities, including BCP-ALL^54^ advocates mechanistic investigation of the circPVT1-miR-497 axis in these leukemias.

CircHIPK3 is a conserved and highly expressed circRNA. Previous studies showed a cytoplasmic location for circHIPK3 and indicated that it is unlikely to be translated, albeit containing the canonical AUG of the associated linear transcripts^38^. CircHIPK3 upregulation was reported in various solid cancers and linked to the reduction of the suppressive activity of several miRNAs^43^, with potential functional implications. CircHIPK3 binding to miR-124 was indeed able to restore expression of miRNA targets sustaining cell proliferation in lung cancer and hepatocellular carcinoma^55,56^. In light of available data, upregulation of circHIPK3 could influence the growth of leukemic cells as well.

The upregulation of circPAX5 in leukemic cells is a new finding, which is worth investigating further to clarify if circPAX5 plays a role in normal haematopoiesis, and explore possible involvement in the disease mechanism. The circPAX5 specific expression in B-cells could point at a cooperating role with the linear transcript that codes for a key B-lineage transcription factor. Predicted binding sites for miRNAs on circPAX5 (Fig. 3b and Supplementary Fig. 9) included miR-124, for which circHIPK3 acts as a sponge^55,56^, and suggested a possible synergy of circRNAs upregulated in leukemic cells. CircPAX5 presented a translation start site in exon 3 wuthouth stop codons afterwards to interrupt the ORF spanning the backsplicing.

Expression of the B-cell specific circAFF3 increased around four times in BCP-ALL. CircAFF3 has putative recognition motifs for several miRNAs, including miR-296–5p, an antimetastatic miRNA whose sequestration by another circRNA was previously linked to cancer^57,58^. In addition, multiple putative binding sites for members of the serine/arginine-rich splicing factor family linked to leukemogenesis^58^, and a 215 aa ORF were predicted for circAFF3 (Fig. 3b and Supplementary Fig. 9).

In conclusion, our data advanced the knowledge of the circular transcriptome in normal hematopoiesis and, besides encouraging future in depth investigation of circRNAs in even more defined sub-populations and blood cell differentiation and maturation stages, may be useful for mechanistic studies of circRNAs in blood cell biology. CircRNA deregulation in patient-derived BCP-ALL samples disclosed by our targeted analysis definitely advocates a multi modal strategy of leukemia transcriptome investigation that integrates linear, circular and miRNAs, instructing also functional investigation of specific circRNAs.

## Methods

### Sample collection, cell sorting and sequencing

Mononuclear cells (PBMCs) were isolated from peripheral blood of healthy adult donors (Department of Transfusional Medicine, University Hospital of Padova; Supplementary Table 1**)** with Lymphoprep^TM^ density gradient centrifugation (STEMCELL Technologies, Vancouver, BC, Canada) followed by haemolysis (0.5 M EDTA pH 8.0). CD45+ (ECD conjugated, Beckman Coulter, Indianapolis, USA) population from PBMCs was separated by sorting with FACS AriaTM III (Becton Dickinson, NJ07417, USA) into B-cells, T-cells and monocytes, after labeling with anti CD19 (clone J4.119, PE-Cy7 conjugated, Beckman Coulter, Indianapolis, USA), CD3 (clone SK7, APC Cy7 conjugated, Becton Dickinson, San Jose, CA, CD14 antibodies) and CD14 (clone RM052, PE conjugated, Beckman Coulter, Indianapolis, USA), respectively. For each cell type, four biological replicates were obtained from different donors.

Total RNA was extracted with TRIzol reagent (Thermo Fisher Scientific, Waltham, Massachusetts) and precipitated with isopropanol. All samples showed a RNA integrity number (RIN) > 7, assessed with Agilent 2100 Bioanalyzer (Santa Clara, CA, USA). RNA libraries were prepared with the TruSeq Stranded Total RNA Ribo-Zero Gold kit. Paired-end reads 100–125 bp long were sequenced with an Illumina® HiSeq2000 (San Diego, CA, USA) at an average sequencing depth of 147 million reads per sample (8 453.25 million bases; Supplementary Table 1). All experiments involving human material followed the principles outlined in the Helsinki Declaration of 1964, as revised in 2000, and informed consent has been obtained for all the participants. The study has been approved by the ethics committee of Padova University Hospital and written informed consent was obtained from all subjects.

### CircRNA detection and quantification

The analysis was based on the Ensembl GRCh38 human genome and annotation v87.

CircRNAs were detected and quantified by CirComPara v0.6^25^ using 9 backsplice detection methods (CIRI2 v2.0.2^26^ Findcirc v1.2^27^; CIRCexplorer2 v2.3.3^28^ combined to each of BWA^29^, STAR^30^, Segemehl^31^ and TopHat2^32^ alignments; DCC v0.4.6^33^; circRNA_finder v1.1^34^; and Segemehl v0.3.4) and default parameters, which selected only the circRNAs detected by two or more methods.

Version of other software tools included in CirComPara: Bowtie2 v2.2.9^59^, BWA v0.7.15-r1140^29^, STAR v2.6.1d^30^, Segemehl v0.3.4^31^, and TopHat2 v2.1.0^32^ with Bowtie v1.1.2^60^.

CirComPara preprocessed raw reads with Trimmomatic v0.38^61^ to remove residual adapters and select reads by quality and length. Read linear mapping to the human genome was performed with HISAT2 v2.0.4^62^.

CirComPara implements circular to linear ratio as described in^33^.

CirComPara’s non-default parameters used for analyses: Adapter sequence = Trimmomatic file TruSeq3-PE.fa; PREPROCESSOR = Trimmomatic, TOGGLE_TRANSCRIPTOME_RECONSTRUCTION = ‘False’, LIN_COUNTER = ‘ccp’, CIRCRNA_METHODS = “testrealign, dcc, ciri, circexplorer2_star, findcirc, circexplorer2_segemehl, circexplorer2_bwa, circexplorer2_tophat, circrna_finder”; PREPROCESSOR_PARAMS = “MAXINFO:40:0.5 LEADING:20 TRAILING:20 SLIDINGWINDOW:4:30 MINLEN:50 AVGQUAL:30”; HISAT2_EXTRA_PARAMS = “–rna-strandness RF “; BWA_PARAMS = [‘-T’, ‘19’, ‘-c’, ‘1’]; SEGEMEHL_PARAMS = [‘-M’,‘1’, ‘-D’, ‘0’]; TOPHAT_PARAMS = [‘–zpacker’, ‘pigz’, ‘–max-multihits’, ‘1’]; STAR_PARAMS = [‘–outFilterMultimapNmax’, ‘1’, ‘–outSJfilterOverhangMin’, ‘15’, ‘15’, ‘15’, ‘15’, ‘–alignSJoverhangMin’, ‘15’, ‘–alignSJDBoverhangMin’, ‘15’, ‘–seedSearchStartLmax’, ‘30’, ‘–outFilterScoreMin’, ‘1’, ‘–outFilterMatchNmin’, ‘1’, ‘–outFilterMismatchNmax’, ‘2’, ‘–chimSegmentMin’, ‘15’, ‘–chimScoreMin’, ‘15’, ‘–chimScoreSeparation’, ‘10’, ‘–chimJunctionOverhangMin’, ‘15’]; MIN_READS = 2; MIN_METHODS = 2; DCC_EXTRA_PARAMS = [‘-fg’, ‘-M’, ‘-F’, ‘-Nr’, 1, 1]; TESTREALIGN_PARAMS = [‘-q’, ‘median_30’]; FINDCIRC_EXTRA_PARAMS = [‘–best-qual’, ‘40’]; FIX_READ_HEADER = ‘True’.

### Differential expression

Differential expression in pairwise comparisons between the three cell types was assessed by DESeq2^63^ (v1.22.2) with local fit, model including sex factor, fold change shrinkage, Wald significance tests, no independent filtering and *poscounts* normalization. P-values were corrected for multiple tests with the Benjamini Hochberg procedure considering at once all the tests performed in all the contrasts. Adjusted p-values ≤ 0.05 were chosen to select the significantly differentially expressed circRNAs.

CircRNA to host-gene linear expression variation was assessed using CircTest^33^ v0.1.1 and selecting adjusted p-values ≤ 0.05.

Circular to linear expression proportion (CLP) for each circRNA was computed as in^33^:

$${\rm{CLP}}=\frac{circular\,reads}{circular\,reads+linear\,reads}$$

Graphics were generated with the ggplot2 v3.1.0, pheatmap v1.0.12, VennDiagram^64^ v1.6.20, and data.table v1.11.8 R packages.

### Experimental validations

Validation of 21 circRNAs was performed on pooled total or RNase R treated RNA of PBMCs from 4 healthy donors to test resistance to RNase R treatment. For 15 of the validated circRNAs qRT-PCR quantification in B-cells, T-cells and monocytes from 5 healthy donors, isolated as described above, was obtained. Moreover, 13 circRNAs were quantified in patient-derived xenograft samples of pediatric BCP-ALL obtained as previously described^65^.

Leukemia samples were obtained from pediatric BCP-ALL patients at diagnosis or relapse upon informed consent of patients and/or their legal guardians in accordance with the institution’s ethical review boards. All animal experiments were approved by the appropriate authority (Regierungspräsidium Tübingen) and carried out following the national animal welfare guidelines.

Total RNA was isolated by TRIzol™ (Thermo Fisher Scientific) extraction, followed by isopropanol precipitation. PBMC RNA was treated with 4 u RNase R/µg (Epicentre) at 37 °C for 15 min and with 5 µl of DNase I (Zymo Research) at room temperature for 15 min. RNA was then purified with RNA Clean & Concentrator ™ −5 (Zymo Research) and quantified with Nanodrop. Reverse Transcription was performed from 500 ng RNA with SuperScriptII (Thermo Fisher Scientific), with random primers (Thermo Fisher Scientific). Divergent primers (Supplementary Table 3) for selective amplification of circRNAs were designed with Primer3 v. 0.4.0^66,67^. RT-PCR was performed from 12.5 ng cDNA with Taq DNA Polymerase (Qiagen) with the following protocol. Initial denaturation: 95 °C for 15 min; 35 cycles: 95 °C for 30 sec, 54–60 °C for 30 sec, 72 °C for 30 sec; final extension: 72 °C for 10 min. Sanger Sequencing was performed on PCR products after cleaning with Wizard® SV Gel and PCR Clean-Up System (Promega), by Eurofins Genomics.

qRT-PCR was performed with technical triplicates with SsoAdvanced Universal SYBR Green Supermix (BioRad) in 10 µl per well, from 5 ng cDNA, 500 nM primers. The reaction was incubated in CFX Connect Real-Time PCR Detection System (BioRad) with the following protocol: 95 °C for 30 sec; 40 cycles: 95 °C for 5 sec, 58 °C for 30 sec. C_q_ were calculated with CFX Maestro™ Software (BioRad). Relative expression is expressed as 2^−∆Cq^ (sample)/average 2^−∆Cq^ (B-cells), where ∆C_q_ = C_q_ (target gene) - Cq (reference gene: B2M). B2M was chosen with NormFinder^68^ as reference gene among three tested (B2M, GAPDH, HPRT).

Statistical significance of differential expression was calculated for comparisons between B-cells, T-cells and monocytes and BCP-ALL vs B-cells with Mann Whitney U-test; for multiple comparison of different genetic subgroups with Kruskal-Wallis test, corrected with the Benjamini, Krieger and Yekuteli procedure. Tests were performed with Prism v7 (GraphPad).

### Functional predictions

Putative circAFF3 and circPAX5 sequences were assembled joining annotated exons comprised in the genomic coordinates corresponding to the validated backplice ends.

MiRNA binding sites were predicted using miRanda^69^, and only target sites with a score in the highest 10% and energy in the lower 10% were considered. Recognition motifs for RNA binding proteins were predicted using beRBP^70^, keeping only sequence motifs with voteFrac score in highest 10%.

Open reading frames of at least 150 nt were predicted using ORFfinder^71^ further keeping only the longest ORF spanning the backsplice.

## Supplementary information

Supplementary Results

Supplementary Table 2

Supplementary Table 5

Supplementary Table 7
