# Supplementary material for: Circular RNA differential expression in blood cell populations and exploration of circRNA deregulation in pediatric acute lymphoblastic leukemia
Source: Sci Rep. 2019 Oct 11;9:14670. doi: 10.1038/s41598-019-50864-z (PMC6789028; doi:10.1038/s41598-019-50864-z)
Supplement: Supplementary file 1 — Supplementary Results [file 41598_2019_50864_MOESM1_ESM.docx]

Circular RNA differential expression in blood cell populations and exploration of circRNA deregulation in pediatric acute lymphoblastic leukemia

Enrico Gaffo, Elena Boldrin, Anna Dal Molin, Silvia Bresolin, Annagiulia Bonizzato, Luca Trentin, Chiara Frasson, Klaus-Michael Debatin, Lueder H. Meyer, Geertruij te Kronnie and Stefania Bortoluzzi

# Supplementary Results

[**Supplementary Results**](#_oyvpr7mzkki5) 1

[**Supplementary Figures**](#_3znysh7) 2

[Supplementary Figure 1. CircRNA predictions shared by methods.](#_2et92p0) 2

[Supplementary Figure 2. Comparison of our results with data of Nicolet et al. 1.](#_6g6sfirjjbn8) 3

[Supplementary Figure 3. Five circRNAs from a new gene mapping at X:65046176-65163942 region of Xq11.2.](#_v236gvem5jpn) 5

[Supplementary Figure 4. qRT-PCR validation of expression of 15 circRNAs in monocytes (M), T-cells (T), and B-cells (B).](#_2s8eyo1) 6

[Supplementary Figure 5. CircRNAs expressed from the PAX5 locus.](#_3rdcrjn) 7

[Supplementary Figure 6. Concordant significant differential expression of circRNAs and circular to linear expression proportion (CLP) in three cell types.](#_lnxbz9) 8

[Supplementary Figure 7. CircSMARCA5 (4:143543509-143543972) expression compared to host-gene linear expression.](#_44sinio) 9

[Supplementary Figure 8. CircRNA expression in BCP-ALL patient-derived xenograft samples.](#_3j2qqm3) 10

[Supplementary Figure 9. Prediction of possible functions and interactions of circAFF3 and circPAX5.](#_1y810tw) 10

[**Supplementary Tables**](#_cqwq4ndzwnij) 12

[Supplementary Table 1. RNA-seq dataset description.](#_1ci93xb) 12

[Supplementary Table 2. Annotation and expression data for the 6,228 high confidence circRNAs identified by at least two methods in the 12 samples of B-cell, T-cell and Monocyte populations and expressed in all replicates of at least one cell type.](#_2bn6wsx) 13

[Supplementary Table 3. Primers and conditions used for qRT-PCR assays on selected circRNAs and linear transcripts.](#_qsh70q) 13

[Supplementary Table 4. The 14 circRNAs with prevalent expression from host-genes (CLP > 0.95) in at least one cell type.](#_49x2ik5) 15

[Supplementary Table 5. CircRNAs differentially expressed comparing B-, T-cell and monocyte populations.](#_147n2zr) 15

[Supplementary Table 6. KEGG pathways, Gene Ontology Biological processes significantly enriched (EnrichR Adj. p-value <0.05) in genes with cell type-specific upregulated circRNAs.](#_pafijuaanvh) 16

[Supplementary Table 7. CircRNAs with significant variation of circular expression proportion and host-gene independent expression.](#_o054ho20sl4) 17

[Supplementary Table 8. Data on healthy subjects and patients screened for circRNA expression by qRT-PCR.](#_32hioqz) 18

[**References**](#_dzdmcw87idyj) 21

# Supplementary Figures

## Supplementary Figure 1. CircRNA predictions shared by methods.

CircRNA expression (read counts in vertical axis) by the number of methods jointly detecting the circRNAs (horizontal axis). Box width is proportional to the number of circRNAs.


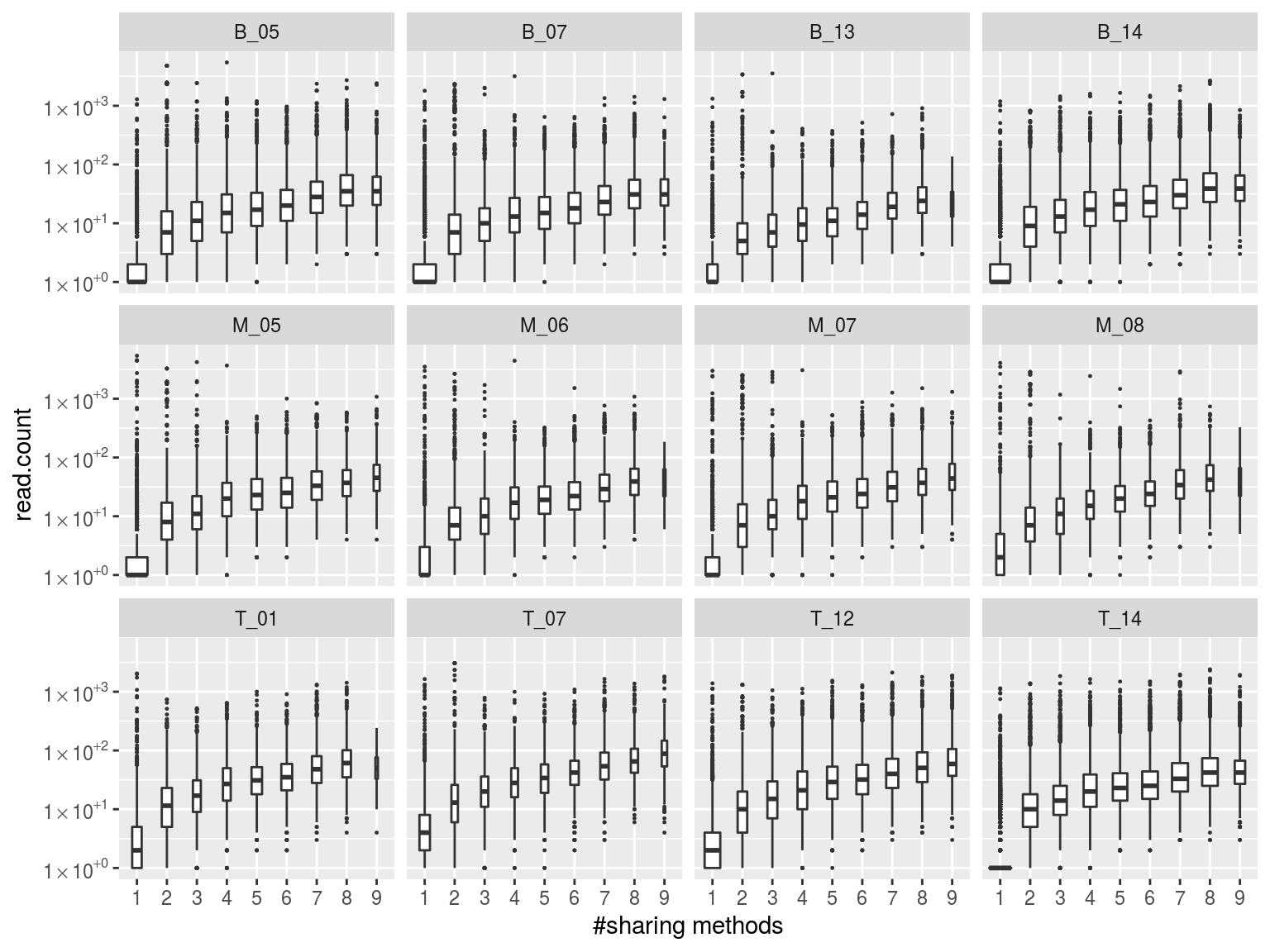


##

##

##

## Supplementary Figure 2. Comparison of our results with data of Nicolet et al. [^1^](https://paperpile.com/c/FvKvEp/LzLb).

Nicolet et al.[^1^](https://paperpile.com/c/FvKvEp/LzLb) considered relatively low depth RNA-seq data in two or three replicates for each of 14 cell populations (Hematopoietic stem cell, Multipotent progenitor, Lymphoid-primed multipotent progenitor, Common lymphoid progenitor, Common myeloid progenitor, Granulocyte-macrophage progenitor, Megakaryocyte-erythrocyte progenitor, CD4+ T cells, CD8+ T cells, Natural killer cells, Monocytes, Granulocytes, Megakaryocytes, and Erythroblasts) obtained with different library construction methods (GSE74912). The present study grounded on high-depth ribodepleted RNA-seq in four replicates of B-, T-cells and monocytes populations (GSE110159). In both studies circRNAs found in all replicates of at least one cell type were deemed “high confidence” (HC), whereas the remaining circRNAs were considered “low confidence” (LC). A) According to the data of the present study, 404 (83%) of the circRNAs previously deemed as HC by Nicolet et al. could be confirmed, whereas 94% (5,824) of the HC circRNAs identified here were not detected in the previous study. B) Expression in our dataset of 98 out of 102 circRNAs previously reported as differentially expressed among blood cell types. Expression of each circRNA is reported as normalized read counts average per cell type, in log_10_ scale; CircRNAs are grouped according to the clusters defined in Nicolet et al.; Cluster labels are reported along with the cluster size and the number of cell type specific circRNAs detected as a fraction (between parenthesis); CircRNAs are identified with host-gene name, circRNA ID; Differential expression significance is indicated within parentheses (NS: not significant overexpression; B: overexpressed only in B-cells; T: overexpressed only in T-cells; M: overexpressed only in monocytes; B+T: overexpressed both in B-cells and T-cells; T+M: overexpressed both in T-cells and monocytes; →: circRNA with differential expression confirmed by qRT-PCR in the present study).

| **a** | **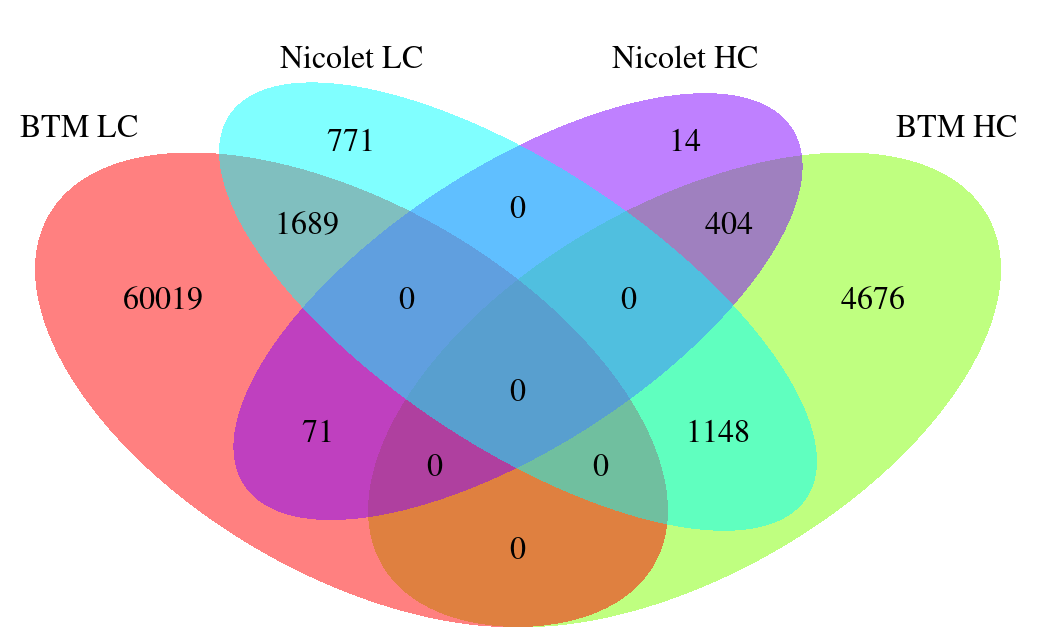** |
| --- | --- |
| **b**  **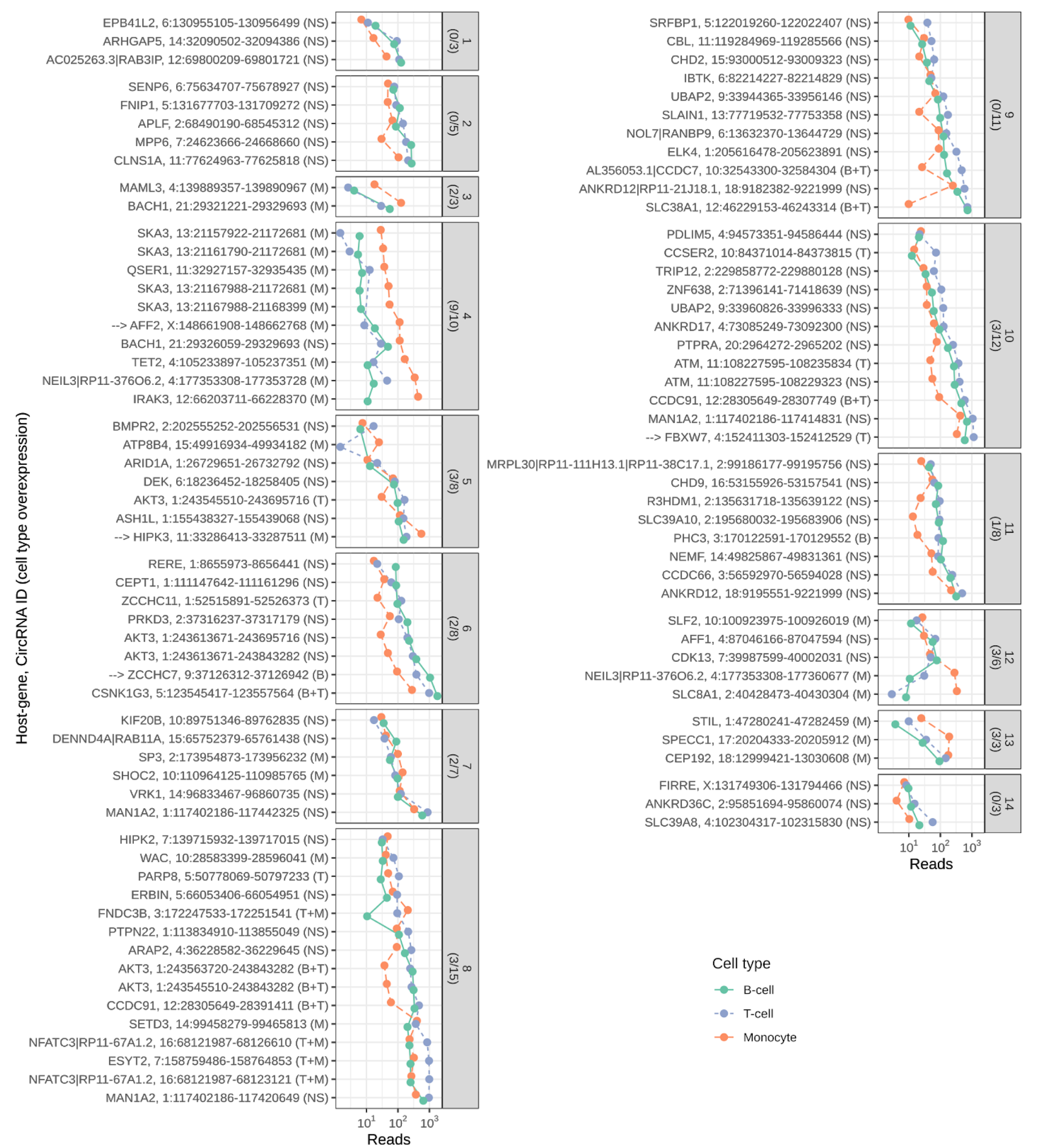** | |

## Supplementary Figure 3. Five circRNAs from a new gene mapping at X:65046176-65163942 region of Xq11.2.

**a)** Representation of the five backsplices mapping at region X:65046176-65163942 of Xq11.2. Average expression levels (normalized reads) in the three cell types are shown in the heatmap. Red color highlights the most expressed circRNA, circX(intergenic)(X:65051462-65075912), which was validated **b)** by RT-PCR on total RNA, RNase R treated RNA and genomic DNA from PBMCs of healthy controls. PCR products of circX(intergenic) were sequenced **c)** confirming the backsplice region (joined nucleotides in bold) and **d)** obtaining a reconstruction of the complete circRNA sequence (blue, red and black colors indicate the three distinct exons).


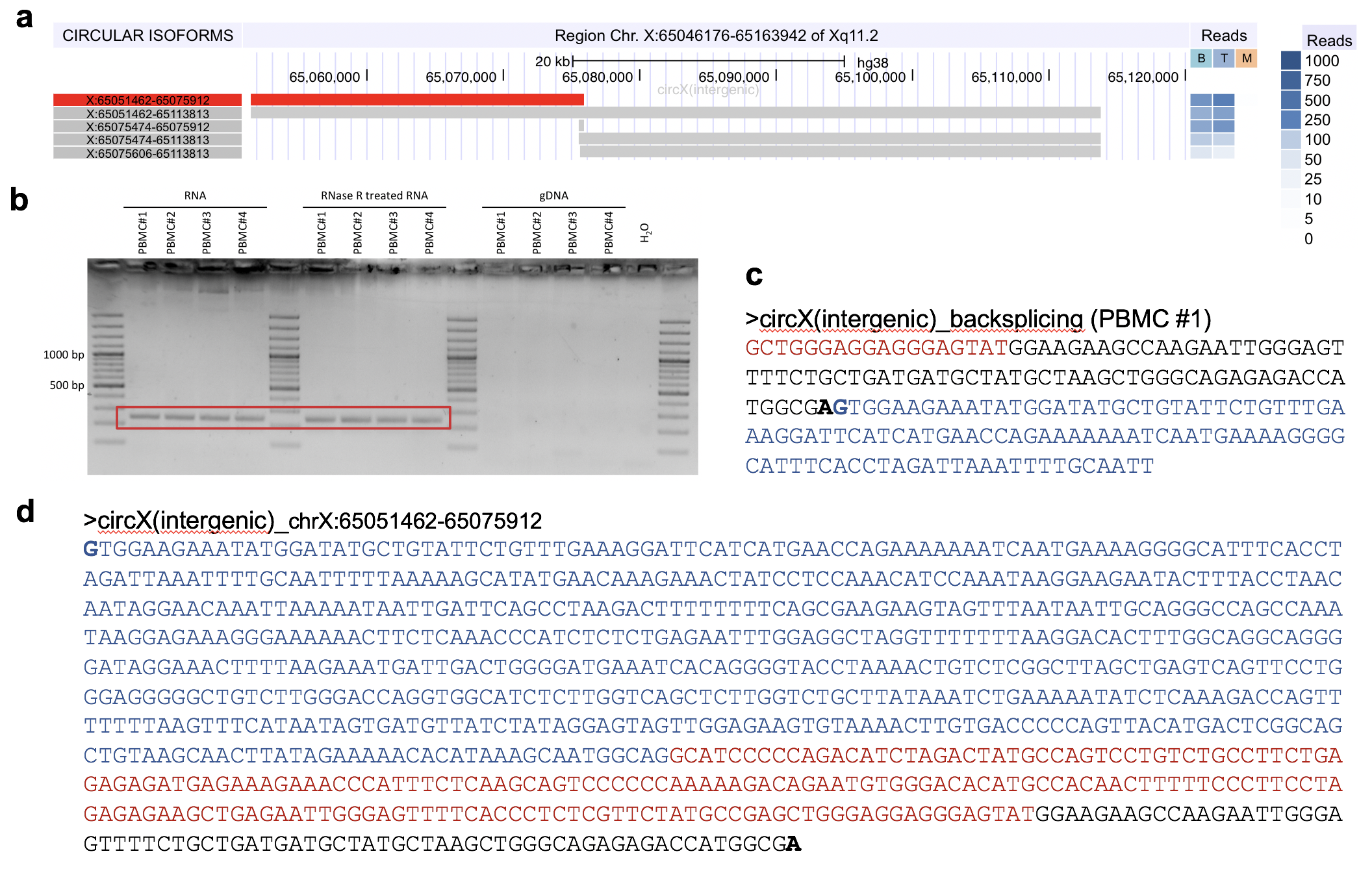


##

## Supplementary Figure 4. qRT-PCR validation of expression of 15 circRNAs in monocytes (M), T-cells (T), and B-cells (B).

Expression relative to the mean of B-cells; Mann Whitney U-test: significant p-values (<0.05) are shown in black, whereas grey values indicates non-significant differences.


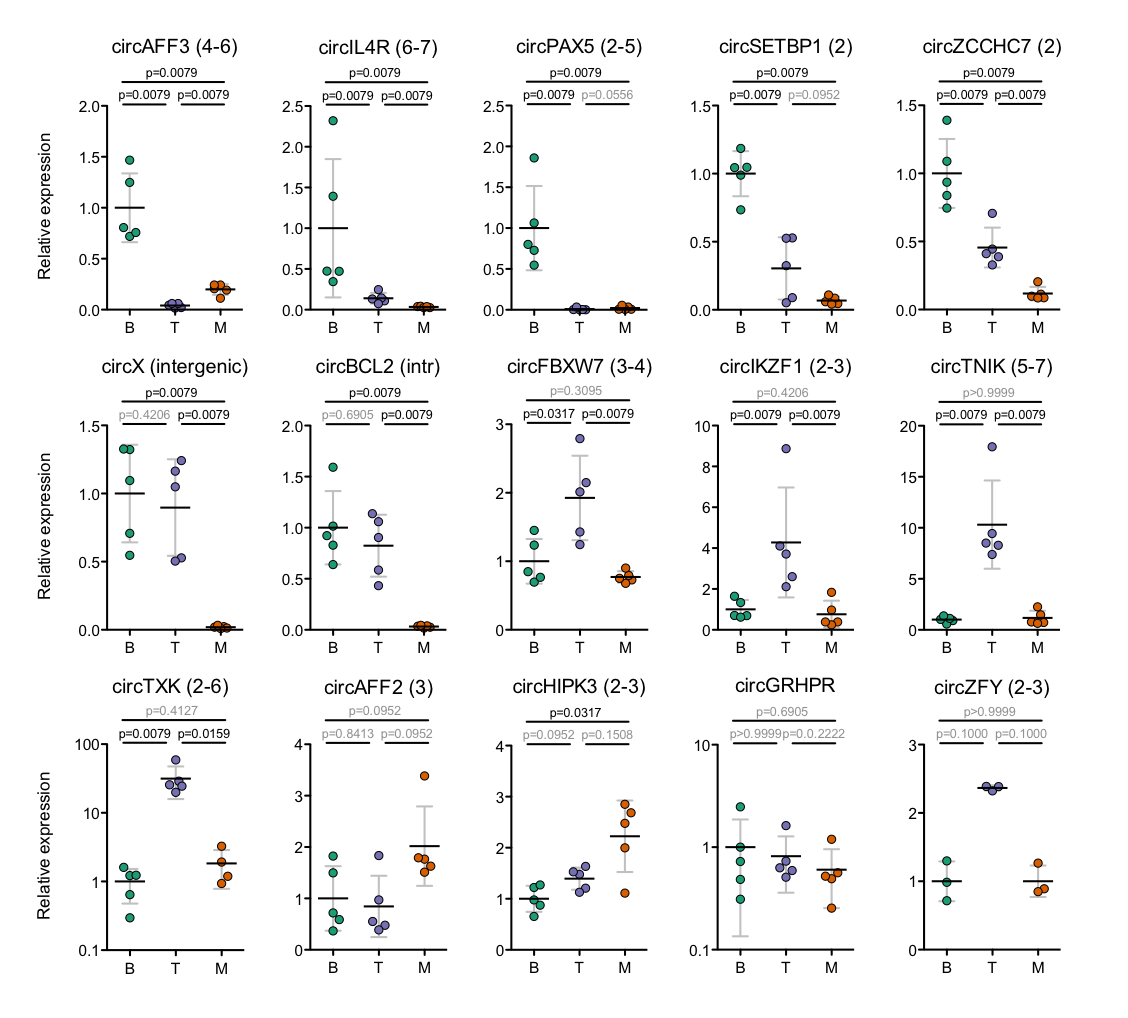


##

## Supplementary Figure 5. CircRNAs expressed from the PAX5 locus.

Validation of PAX5, ZCCHC7 and GRHPR circRNAs mapping on chromosome 9 (region 9:36923355-37432138 of 9p13.2). **a)** CircRNAs mapping on chr. 9:36923355-37432138 of 9p13.2. Average expression levels (normalized reads) in the three cell types are shown on the heatmap. Colors indicate the most expressed circPAX5(2-5)(9:37002645-37020798; red), circZCCHC7(2)(9:37126308-37126939; green), circGRHPR(2-4)(9:37424842-3742665; orange). Validation by RT-PCR on total RNA, RNase R treated RNA and gDNA from PBMCs of healthy controls of **b)** circPAX5, **c)** circZCCHC7 and **d)** circGRHPR. **e)** Sequence of the PCR product of circPAX5 (3´of exon 5 in black and 5´of exon 2 in blue). Sequences of the lower band in **f)** of circZCCHC7 (3´of exon 2 in black, followed by 5´of exon 2 in blue); **f)** upper band of circZCCHC7 (3´of exon 2 in black, and an intronic sequence in gray); **h)** lower band of circGRHPR (3´of exon 4 in black and 5´of exon 2 in blue); **i)** lower band of circGRHPR. The backsplice junction in **i)** is the same as the one identified by the sequence of the lower band in **h)**, but this amplicon, generated upon rolling circle amplification, revealed the structure of the circRNA, with the 3´of exon 2 of GRHPR (blue) followed by exon 3 (gray) and 4 (black) and the 5´of exon 2 (blue).


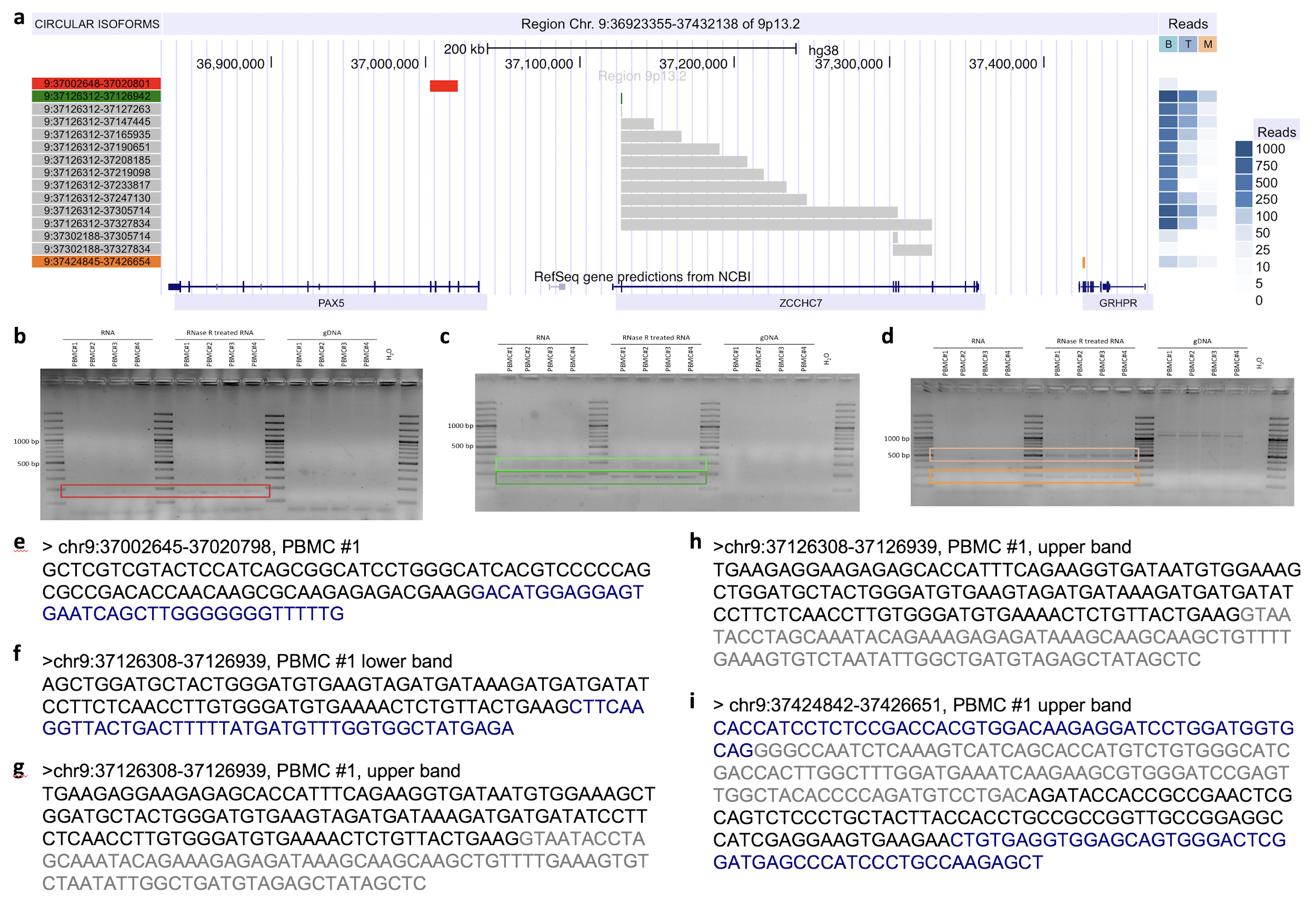


## Supplementary Figure 6. Concordant significant differential expression of circRNAs and circular to linear expression proportion (CLP) in three cell types.

CircRNA names on the right side are composed as “host-gene name @ genomic coordinates of circRNA backplice ends” (hg38). Heatmap color bar shows circRNA expression and median CLP in each cell type as shown on the left. The heatmap shows the 25 circRNAs that contribute to over two thirds of host-gene total read count.

##
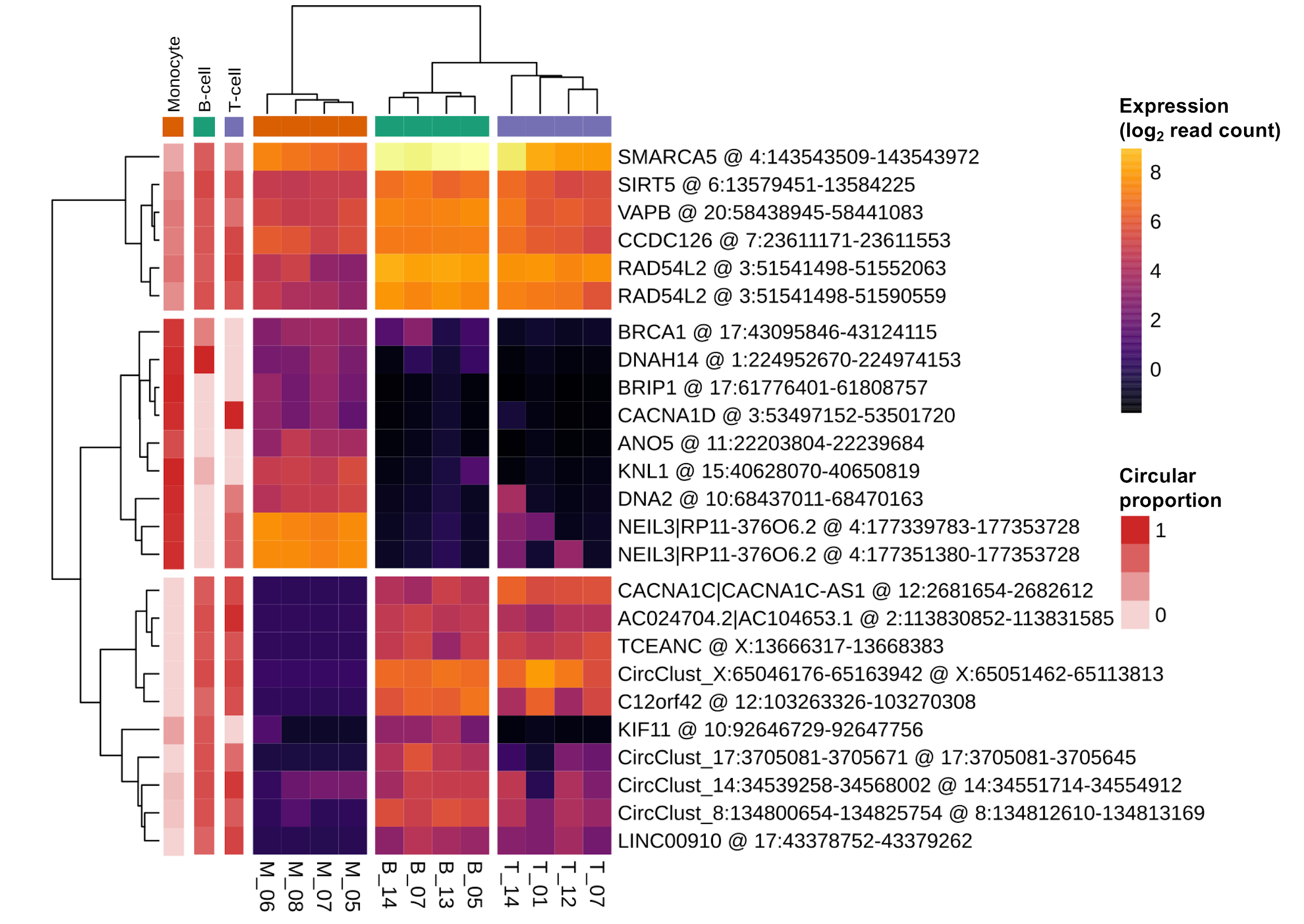


##

## Supplementary Figure 7. CircSMARCA5 (4:143543509-143543972) expression compared to host-gene linear expression.

**a)** Mean circular to linear expression proportion in B-cells, T-cells and monocytes; **b)** Circular and linear read count.

**a** **b**

circSMARCA5 circSMARCA5

##
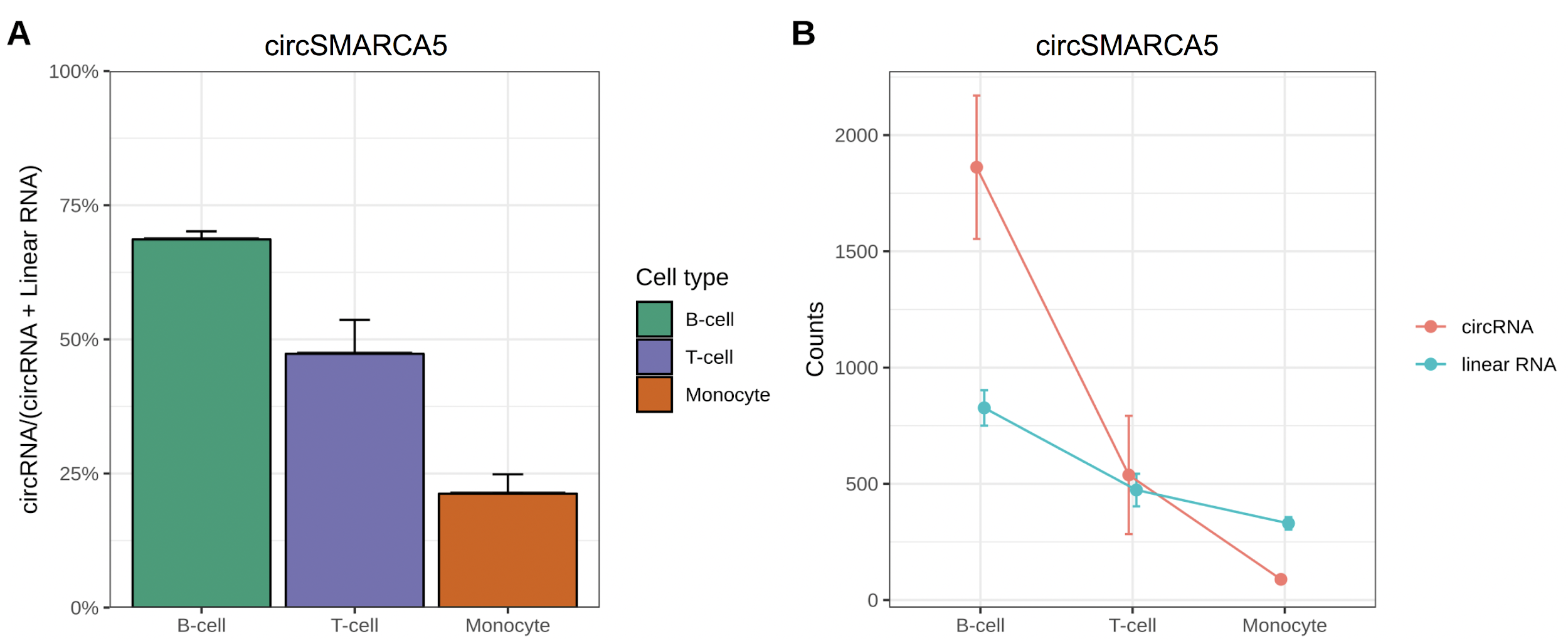


##

## Supplementary Figure 8. CircRNA expression in BCP-ALL patient-derived xenograft samples.

Expression of 13 circRNAs assessed by qRT-PCR in B-cells and 32 BCP-ALL patient-derived xenograft samples. Expression relative to B-cells; Mann Whitney U-test: significant p-values (<0.05) are shown in black, whereas grey values indicate non-significant differences.


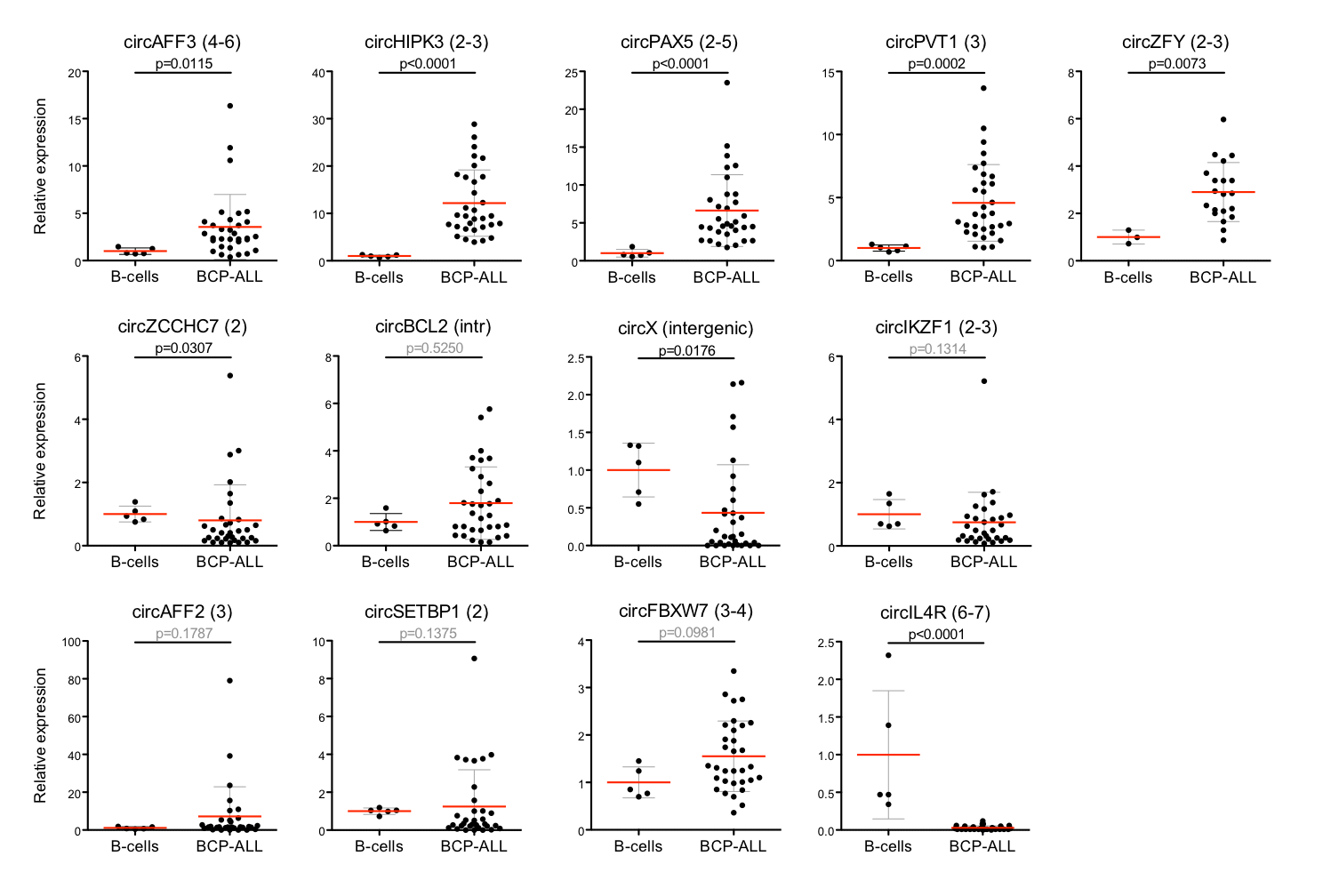


## Supplementary Figure 9. Prediction of possible functions and interactions of circAFF3 and circPAX5.

For each of the circRNAs, predicted miRNA and RNA binding protein (RBP) binding sites, and the longest open reading frame spanning the backplice are indicated; Nucleotide positions of ORFs refer to the circRNA sequence considered two times; Regions of the predicted peptides corresponding and not corresponding to the canonical protein encoded by the gene are shown in black and blue, respectively, and methionines are shown in red.


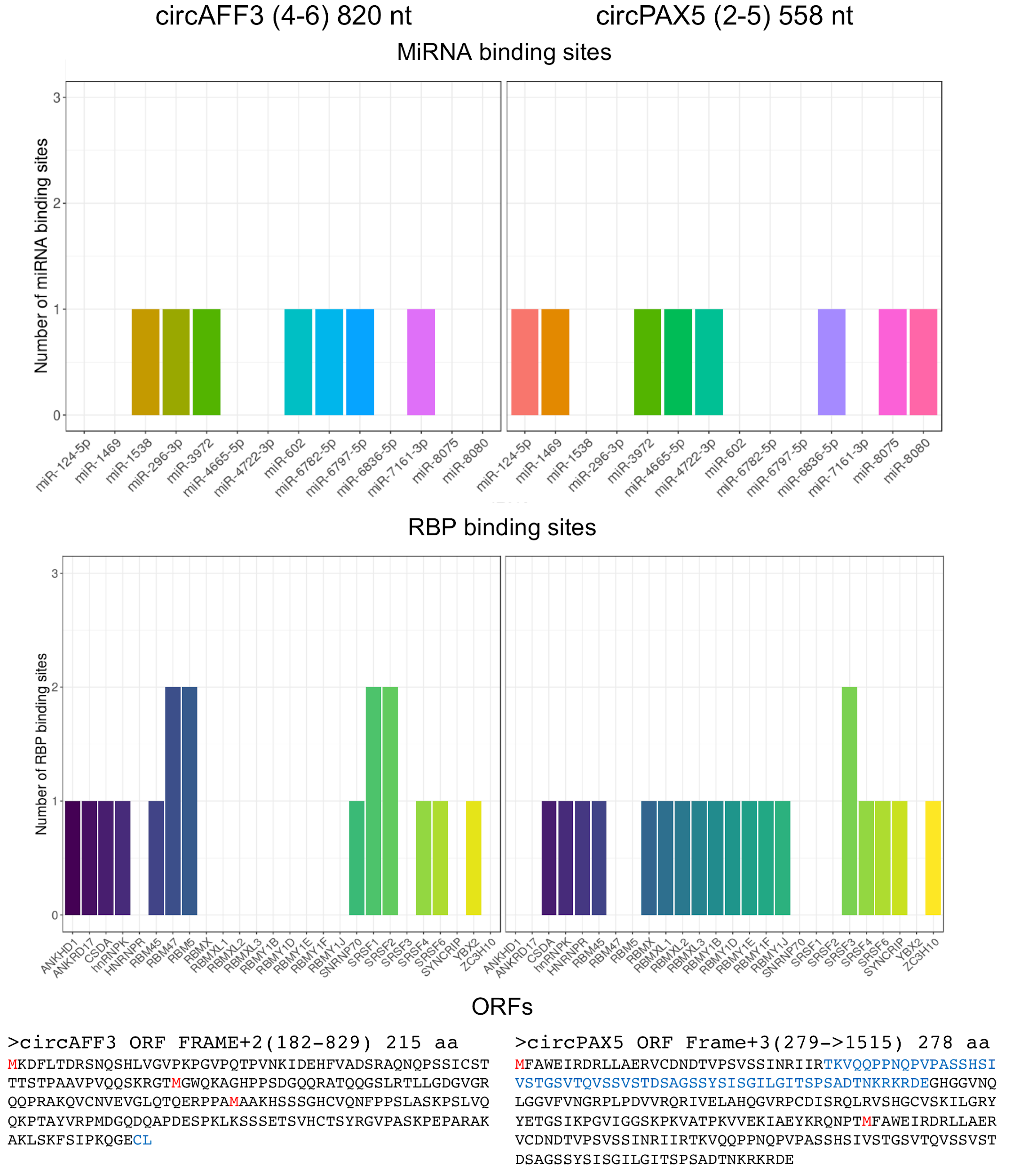


# Supplementary Tables

##

## Supplementary Table 1. RNA-seq dataset description.

For each sample, data regarding donors, cell type, RNA quality and concentration, and sequencing depth are indicated.

| **Sample** | **Cell type** | **Sex** | **Sorted cells (millions)** | **RIN** | **RNA (ng/μl)** | **Raw reads** | **Sequenced bases** |
| --- | --- | --- | --- | --- | --- | --- | --- |
| b05 | CD19+ | M | 3.9 | 7.3 | 43.4 | 118585032 | 6967439400 |
| b07 | CD19+ | M | 4.5 | 7.8 | 110.0 | 129235360 | 7582930150 |
| b13 | CD19+ | M | 3.9 | 7.8 | 69.9 | 108236716 | 5411835800 |
| b14 | CD19+ | F | 2.3 | 8.5 | 188.0 | 142565832 | 8372294850 |
| m05 | CD14+ | M | 3.4 | 9.1 | 95.6 | 192086930 | 12005433125 |
| m06 | CD14+ | F | 4.8 | 7.8 | 76.2 | 146374148 | 7318707400 |
| m07 | CD14+ | M | 4.5 | 8.0 | 99.4 | 124539144 | 7783696500 |
| m08 | CD14+ | M | 4.8 | 9.0 | 1180.0 | 131493682 | 6574684100 |
| t01 | CD3+ | F | 7.8 | 8.6 | 63.6 | 166967662 | 8348383100 |
| t07 | CD3+ | M | 5.0 | 8.3 | 151.0 | 172067828 | 10754239250 |
| t12 | CD3+ | M | 7.5 | 8.4 | 232.0 | 182155352 | 11384709500 |
| t14 | CD3+ | F | 2.5 | 8.4 | 306.0 | 151858242 | 8934619475 |

## Supplementary Table 2. Annotation and expression data for the 6,228 high confidence circRNAs identified by at least two methods in the 12 samples of B-cell, T-cell and Monocyte populations and expressed in all replicates of at least one cell type.

(see separate PDF file).

## Supplementary Table 3. Primers and conditions used for qRT-PCR assays on selected circRNAs and linear transcripts.

| **CircRNA**  **(name**  **ID**  **CircBase ID)** | **Gene** | **Transcript** | **Primer forward** | **Primer reverse** | **Annealing T RT-PCR (°C)** | **Annealing T qRT-PCR (°C)** | **Amplicon length** | **qRT-PCR efficiency** |
| --- | --- | --- | --- | --- | --- | --- | --- | --- |
| circAFF2 (3)  X:148661908-148662768:+  hsa_circ_0001947 | AFF2 | NM_002025.3 | TGGAACACTCTTGGATGGAAA | CATTGGTGCTGAAGGATGG | 58 | 58 | 310 | 93.4 |
| circAFF3 (4-6)  2:100006632-100008932:-  hsa_circ_0116882 | AFF3 | NM_002285.2 | TGAGTCTCCTAAGCTGAAGTCGT | GTGCCATCATCCTGTTGAGTT | 58 | 58 | 217 | 89.8 |
| circBCL2 (intr)  18:63280887-63281214:-  hsa_circ_0141408\|hsa_circ_0108783 | BCL2 | - | TGGATACTTGAGCTGCTTCC | TGAGTCCACTTATGCGAGGT | 58 | 58 | 181 | 96.3 |
| circCSF2RA (4-7)  X:1285778-1290509:+  NA | CSF2RA | NM_006140.4 | CGTCTCGCAATTACTTTCTGG | CTGGGTTCCACGACTCTGTT | 52 | 58 | 229 | - |
| circFBXW7 (3-4)  4:152411303-152412529:-  hsa_circ_0001451\|hsa_circ_001988 | FBXW7 | NM_001257069.1 | CTGCCCGTTCACCAACTC | TGCTGTTCCTCCTCTACCACA | 60 | 58 | 292 | 101.7 |
| circGRHPR (2-4)  9:37424845-37426654:+  hsa_circ_0001861 | GRHPR | NM_012203.1 | AGATACCACCGCCGAACTC | GGAGAGGAGGCAGAGCAG | 58 | 58 | 178 | 116.6 |
| circHIPK3 (2)  11:33286413-33287511:+  hsa_circ_0000284 | HIPK3 | NM_005734.5 | GGCAGCCTTACAGGGTTAAAG | CATTCACATAGGTCCGTGGATA | 58 | 58 | 235 | 91.9 |
| circIKZF1 (2-3)  7:50319048-50327757:+  hsa_circ_0001708\|hsa_circ_001191 | IKZF1 | NM_006060.5 | AAGAGTGACAGAGTCGTGGATAAC | TGTCTTGACCCTCATCAGCA | 58 | 58 | 58 | 102.3 |
| circIKZF1 (4-7)  7:50376533-50391863:+  NA | IKZF1 | NM_006060.5 | CAGTGAAATGGCAGAAGACC | CCTCCAACTCCCGACAAA | 52 | 58 | 279 | 91.3 |
| circIL4R (6-7)  16:27346467-27352696:+  hsa_circ_0038724 | IL4R | NM_000418.4 | CCTGAAGTCTGGGATTTCCT | TCGGAGACATTGGTGTGAAC | 58 | 58 | 150 | 95.6 |
| circPVT1 (3)  8:127890589-127890998:+  hsa_circ_0001821 | PVT1 | NR_003367.3 | CTCTTCCTGGTGAAGCATCTG | CTGACAGGCACAGCCATCT | 58 | 58 | 118 | 84.1 |
| circPAX5 (2-5)  9:37002648-37020801:-  hsa_circ_0001857\|hsa_circ_001337 | PAX5 | NM_016734.2 | GCTCGTCGTACTCCATCAGC | AGCAAGTTCCACTATCCTCTGG | 58 | 58 | 167 | 104.9 |
| circSETBP1 (2)  18:44701175-44701832:+  hsa_circ_0006491 | SETBP1 | NM_015559.3 | CACAAAGCGGGCTAAGAAAC | GGTCCAGTTGCTAGGGTCAG | 58 | 58 | 232 | 97.3 |
| circSETD3 (2-6)  14:99458279-99465813:-  hsa_circ_0000567 | SETD3 | NM_032233.2 | AGCCAGCCCTAACTCCTTCT | AGGTTCAAGATTTCCTTTGGTG | 58 | 58 | 272 | 104.2 |
| circTNIK (5-7)  3:171188702-171194635:-  hsa_circ_0002387 | TNIK | NM_015028.4 | TGGAACTCCCTACTGGATGG | TCCACTCCTCTTTCAACGTG | 58 | 58 | 155 | 108.4 |
| circTXK (2-6)  4:48104901-48114402:-  NA | TXK | NM_003328.3 | CCAGAAATCAGGCAGAACATC | GCAACAGCAGCAACAGAAAA | 58 | 58 | 73 | 101.5 |
| circZCCHC7 (2)  9:37126312-37126942:+  hsa_circ_0001860\|hsa_circ_000489 | ZCCHC7 | NM_001289121.1 | TGAAGAGGAAGAGAGCACCA | TCTCATAGCCACCAAACATCA | 60 | 58 | 174 | 97.1 |
| circZFY (2-3)  Y:2953909-2961646:+  hsa_circ_0001953\|hsa_circ_001871 | ZFY | NM_003411.3 | GGACCAGCAAGATAATGACAAA | CCATCCATGTGTGTAGCATCAG | 54 | 58 | 167 | 99.3 |
| circZFY (3)  Y:2961074-2961646:+  hsa_circ_0007907 | ZFY | NM_003411.3 | CAGCGGGATCTCAGTGGAC | TGAGTCTGGGTCATCAGGAA | 52 | 58 | 192 | 101.2 |
| circZNF609 (1)  15:64499293-64500166:+  hsa_circ_0000615 | ZNF609 | NM_015042.1 | GAAGGGGAGAATGAGTGTCG | CGTCCAGGTCAATGATGAGA | 54 | 58 | 166 | 96.9 |
| circX (intergenic)  X:65051462-65075912:+  hsa_circ_0001924 | - | - | GCTGGGAGGAGGGAGTATG | CCTTATTTGGATGTTTGGAGGA | 58 | 58 | 242 | 113.1 |
| - | GAPDH | - | CAGAACATCATCCCTGCCTCTACTGGC | GGTCTTACTCCTTGGAGGCCATGTGGG | - | 58 | - | - |
| - | HPRT | - | GAGATGGGAGGCCATCACATTGTAGCCCTC | CTCCACCAATTACTTTTATGTCCCCTCTTCACTGGTC | - | 58 | - | - |
| - | B2M | - | GTGGAGCATTCAGACTTGTCTTTCAGCAAGGAC | CACTTAACTATCTTGGCCTGTGACAAAGTCACATGG | - | 58 | - | - |

##

## Supplementary Table 4. The 14 circRNAs with prevalent expression from host-genes (CLP > 0.95) in at least one cell type.

| **CircRNA** | **Gene name** | **B-cell**  **median CLP** | **T-cell**  **median CLP** | **Monocyte**  **median CLP** |
| --- | --- | --- | --- | --- |
| 6:26885359-26889197 | GUSBP2 | 1 | 1 | 1 |
| 1:146074271-146111332 | NBPF10 | 0.99 | 1 | 0.99 |
| 4:177353308-177360677 | NEIL3 | 0.96 | 0.89 | 0.96 |
| 4:104518577-104519454 | AC004053.1 | 0.92 | 0.43 | 1 |
| 1:65364635-65366196 | DNAJC6 | 0.72 | 0.68 | 1 |
| 7:87964634-87978412 | ADAM22 | 0.95 | 0.68 | 0 |
| 2:106434637-106435202 | RGPD3 | 0.65 | 0.13 | 1 |
| X:148651999-148662768 | AFF2 | 0.38 | 0.58 | 0.97 |
| 2:40428473-40430304 | SLC8A1 | 0.56 | 1 | 0.54 |
| 10:46794727-46798168 | BMS1P1 | 0.51 | 0.54 | 1 |
| 9:93335374-93335976 | C9orf129 | 0.33 | 1 | 0 |
| X:101126709-101148161 | CENPI | 0 | 0.33 | 1 |
| 20:43702490-43705358 | MYBL2 | 0.13 | 0 | 1 |
| 18:45611491-45611797 | SLC14A2 | 1 | 0 | 0 |

##

## Supplementary Table 5. CircRNAs differentially expressed comparing B-, T-cell and monocyte populations.

(see separate PDF file)

##

## Supplementary Table 6. KEGG pathways, Gene Ontology Biological processes significantly enriched (EnrichR Adj. p-value <0.05) in genes with cell type-specific upregulated circRNAs.

| **Cell type** | **Category type** | **Term** | **Adj. P-value** | **Combined Score** | **Genes with circRNAs expressed with cell type-specificity** |
| --- | --- | --- | --- | --- | --- |
| T-cell | GO BP | positive regulation of antigen receptor-mediated signaling pathway (GO:0050857) | 0.001 | 645 | PRKCH;PTPRC;LCK;SLC39A10;CD226 |
| T-cell | GO BP | protein autophosphorylation (GO:0046777) | 0.047 | 62 | ITK;LCK;TXK;STK39;AAK1;ATM;TNIK;STK4 |
| T-cell | GO BP | phosphorylation (GO:0016310) | 0.047 | 45 | PRKCH;LCK;TXK;AKT3;STK38;NEK7;STK39;AAK1;PRKCA;ATM;TNIK;STK4 |
| T-cell | KEGG | T cell receptor signaling pathway | 0.003 | 110 | ITK;PTPRC;PPP3CC;LCK;AKT3;CBLB;RASGRP1 |
| Monocyte | GO BP | neutrophil mediated immunity (GO:0002446) | 0.001 | 47 | CD53;ACTR2;MANBA;ATP8B4;ITGAM;APAF1;ROCK1;SLC11A1;NFAM1;RAB3D;GDI2;ADAM10;CYBB;IQGAP1;PSEN1;PLD1;CTSS;DNAJC3;ITGAX;PECAM1;CD36;ATG7;CD44 |
| Monocyte | GO BP | neutrophil activation involved in immune response (GO:0002283) | 0.001 | 48 | CD53;ACTR2;MANBA;ATP8B4;ITGAM;APAF1;ROCK1;SLC11A1;NFAM1;RAB3D;GDI2;ADAM10;CYBB;IQGAP1;PSEN1;PLD1;CTSS;DNAJC3;ITGAX;PECAM1;CD36;ATG7;CD44 |
| Monocyte | GO BP | neutrophil degranulation (GO:0043312) | 0.003 | 48 | CD53;ACTR2;MANBA;ATP8B4;ITGAM;APAF1;ROCK1;SLC11A1;NFAM1;RAB3D;GDI2;ADAM10;CYBB;IQGAP1;PSEN1;PLD1;CTSS;DNAJC3;ITGAX;PECAM1;CD36;ATG7;CD44 |
| Monocyte | GO BP | regulation of neuroblast proliferation (GO:1902692) | 0.008 | 327 | LRRK2;NF1;CTNNB1;DISC1 |
| Monocyte | GO BP | interferon-gamma-mediated signaling pathway (GO:0060333) | 0.010 | 92 | HLA-DRB5;IFNGR2;HLA-B;HLA-C;IFI30;JAK2;CD44;HLA-DRB1 |
| Monocyte | GO BP | strand displacement (GO:0000732) | 0.031 | 132 | BRIP1;RBBP8;ATM;BRCA1;DNA2 |
| Monocyte | KEGG | Pathways in cancer | 0.009 | 29 | SPI1;APAF1;ROCK1;FLT3;IFNGR2;NCOA4;LPAR1;PTEN;PLD1;ADCY7;CSF2RA;GNAI2;RXRA;MSH3;SP1;GNAQ;CTNNB1;JAK2;SOS2;IL13RA1;NFE2L2 |
| Monocyte | KEGG | Parathyroid hormone synthesis, secretion and action | 0.009 | 46 | MEF2A;AKAP13;RXRA;SP1;GNAQ;PLD1;ADCY7;GNAI2 |
| Monocyte | KEGG | Antigen processing and presentation | 0.009 | 57 | HLA-DRB5;CANX;HLA-B;HLA-C;IFI30;CTSS;HLA-DRB1 |
| Monocyte | KEGG | Leishmaniasis | 0.009 | 61 | HLA-DRB5;ITGAM;NCF2;IFNGR2;CYBB;JAK2;HLA-DRB1 |
| Monocyte | KEGG | Phagosome | 0.011 | 44 | HLA-DRB5;ITGAM;NCF2;CANX;HLA-B;HLA-C;CYBB;CD36;HLA-DRB1;CTSS |
| Monocyte | KEGG | Hematopoietic cell lineage | 0.026 | 38 | HLA-DRB5;ITGAM;FLT3;CD36;CSF2RA;HLA-DRB1;CD44 |
| Monocyte | KEGG | Notch signaling pathway | 0.027 | 54 | LFNG;NCOR2;NUMB;PSEN1;MAML3 |
| Monocyte | KEGG | Human T-cell leukemia virus 1 infection | 0.044 | 21 | HLA-DRB5;SPI1;XPO1;CANX;HLA-B;PTEN;HLA-C;ATM;ADCY7;HLA-DRB1 |
| Monocyte | KEGG | Leukocyte transendothelial migration | 0.045 | 29 | ITGAM;ROCK1;NCF2;PECAM1;CYBB;CTNNB1;GNAI2 |
| Monocyte | KEGG | Human cytomegalovirus infection | 0.049 | 20 | AKAP13;ROCK1;SP1;GNAQ;HLA-B;HLA-C;CTNNB1;ADCY7;SOS2;GNAI2 |

##

## Supplementary Table 7. CircRNAs with significant variation of circular expression proportion and host-gene independent expression.

CLP is the median proportion of circRNA expression relative to the host-gene linear expression of each cell type, computed as $\frac{circular reads}{(circular reads + linear reads)}$. If a backsplice spans multiple genes, they are reported as a bar separated list. *P-adj* columns report the significant (BH adjusted p-value ≤ 0.05) statistics computed by CircTest.

(see separate PDF file)

## Supplementary Table 8. Data on healthy subjects and patients screened for circRNA expression by qRT-PCR.

| **Sample** | **Group** | **Sex** |
| --- | --- | --- |
| #1 B | B-cells | F |
| #2 B | B-cells | M |
| #3 B | B-cells | M |
| #4 B | B-cells | M |
| #5 B | B-cells | F |
| #1 M | Monocytes | F |
| #2 M | Monocytes | M |
| #3 M | Monocytes | M |
| #4 M | Monocytes | M |
| #5 M | Monocytes | F |
| #1 T | T-cells | F |
| #2 T | T-cells | M |
| #3 T | T-cells | M |
| #4 T | T-cells | M |
| #5 T | T-cells | F |
| PDX1 | Others | M |
| PDX2 | Others | M |
| PDX3 | Others | F |
| PDX4 | Others | M |
| PDX5 | Others | M |
| PDX6 | Others | M |
| PDX7 | Others | M |
| PDX8 | Others | M |
| PDX9 | BCR/ABL | M |
| PDX10 | BCR/ABL | F |
| PDX11 | BCR/ABL | F |
| PDX12 | ETV6-RUNX1 | M |
| PDX13 | ETV6-RUNX1 | F |
| PDX14 | ETV6-RUNX1 | M |
| PDX15 | ETV6-RUNX1 | M |
| PDX16 | ETV6-RUNX1 | M |
| PDX17 | ETV6-RUNX1 | M |
| PDX18 | Hyperdiploid | M |
| PDX19 | Hyperdiploid | F |
| PDX20 | Hyperdiploid | M |
| PDX21 | Hyperdiploid | F |
| PDX22 | Hyperdiploid | F |
| PDX23 | Hyperdiploid | M |
| PDX24 | Hyperdiploid | F |
| PDX25 | MLL | F |
| PDX26 | MLL | F |
| PDX27 | MLL | M |
| PDX28 | MLL | M |
| PDX29 | TCF3-PBX1 | F |
| PDX30 | TCF3-PBX1 | F |
| PDX31 | TCF3-PBX1 | M |
| PDX32 | TCF3-PBX1 | M |

# References

1. [Nicolet, B. P. *et al.* Circular RNA expression in human hematopoietic cells is widespread and cell-type specific. *Nucleic Acids Res.* **46**, 8168–8180 (2018).](http://paperpile.com/b/FvKvEp/LzLb)
